# Structural and Kinetic Profiling of Allosteric Modulation of Duplex DNA Induced by DNA‐Binding Polyamide Analogues

**DOI:** 10.1002/chem.201805338

**Published:** 2019-01-14

**Authors:** Khalid Aman, Giacomo Padroni, John A. Parkinson, Thomas Welte, Glenn A. Burley

**Affiliations:** ^1^ Department of Pure and Applied Chemistry University of Strathclyde, Thomas Graham Building 295 Cathedral Street Glasgow G1 1XL UK; ^2^ Dynamic Biosensors GmbH 82152 Planegg Germany

**Keywords:** allosterism, binding kinetics, minor groove binder, NMR characterisation, pyrrole-imidazole polyamide

## Abstract

A combined structural and quantitative biophysical profile of the DNA binding affinity, kinetics and sequence‐selectivity of hairpin polyamide analogues is described. DNA duplexes containing either target polyamide binding sites or mismatch sequences are immobilized on a microelectrode surface. Quantitation of the DNA binding profile of polyamides containing N‐terminal 1‐alkylimidazole (Im) units exhibit picomolar binding affinities for their target sequences, whereas 5‐alkylthiazole (Nt) units are an order of magnitude lower (low nanomolar). Comparative NMR structural analyses of the polyamide series shows that the steric bulk distal to the DNA‐binding face of the hairpin *i*Pr‐Nt polyamide plays an influential role in the allosteric modulation of the overall DNA duplex structure. This combined kinetic and structural study provides a foundation to develop next‐generation hairpin designs where the DNA‐binding profile of polyamides is reconciled with their physicochemical properties.

## Introduction

**Figure 1:**
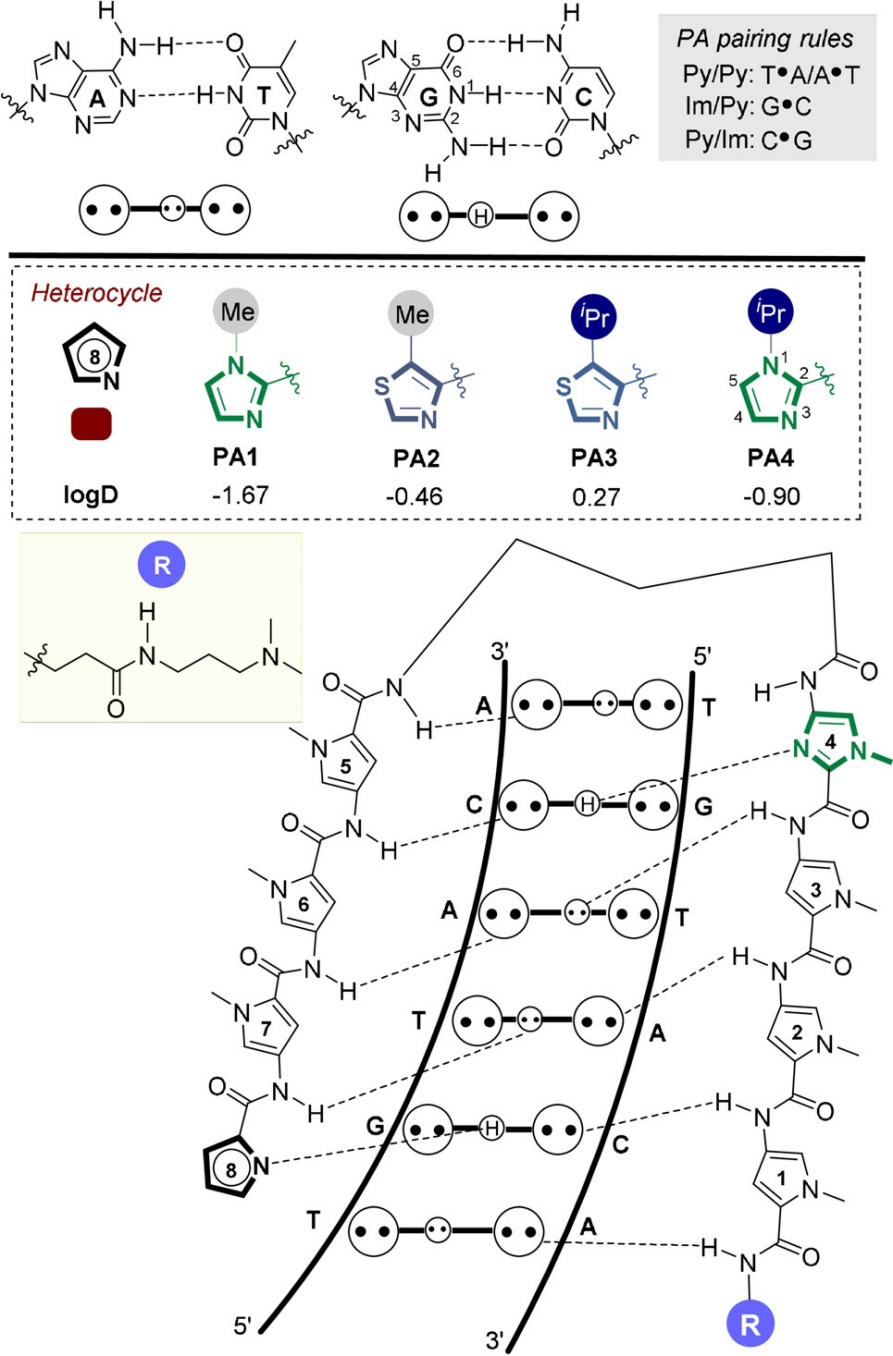
General binding mode of hairpin PAs used in this study.

DNA‐binding polyamides (PAs) are cell‐permeable transcriptional modulators which function by inhibiting RNA polymerase‐mediated elongation and/or transcription factor binding to its target double‐stranded DNA (dsDNA) consensus sequence.^1^ Of the various designs reported,^2^ hairpin PAs are the most widely used1b,1c,1f, 3 where the primary sequence of *N*‐methyl pyrrole (Py) and *N*‐methyl imidazole (Im) heterocyclic amino acids defines the selectivity of dsDNA binding ranging from 7 up to 24 base‐pairs in length (e.g., **PA1**, Figure 1).1b, 4 At present, an unmet challenge in their further development as a general tool to modulate gene‐selective transcription is an in‐depth understanding of the interplay between the dsDNA binding profile of PAs determined in vitro, with their overall physicochemical properties which impact cell uptake, and ultimately target engagement in vivo.^5^

We have recently expanded the heterocyclic repertoire of current Py‐Im hairpin PA designs to include N‐terminal thiazole‐4‐carboxylic acid units (Nt).^6^ Nt‐building blocks (e.g., **PA2**–**3**) direct a hydrogen‐bond‐acceptor (N3) atom towards the floor of the minor groove and forms a hydrogen bond with the exocyclic hydrogen bond donor amine (N2) of G. A key structural difference with the incorporation of an Nt‐unit in the N‐terminal position of a hairpin PA is the endocyclic sulfur atom which changes both the geometry and hydrophobicity (logD) of this heterocycle (Figure 1).^7^ Furthermore, when a bulky isopropyl substituent is installed in the 5‐position (i.e., *i*Pr‐Nt, **PA3**), a more pronounced compression of the major groove is observed relative to the archetypical hairpin **PA1⋅**dsDNA complex.^6^ These results imply that allosteric modulation of the DNA duplex imparted by PAs is influenced by both the nature of the N‐terminal heterocycle pairing with the N2 of G, and the steric bulk of substituents not directly involved in selective minor groove recognition.^8^ What is unclear at present is how these changes to the N‐terminus influence the kinetics of target versus mismatch binding to dsDNA sequences.

In this manuscript, we report a label‐free biophysical assay to profile the affinity, sequence‐selectivity and binding kinetics of PA**⋅**dsDNA interactions where the N‐terminal heterocycle is systematically altered (**PA1**–**4**). PAs containing N‐terminal Im units (i.e., **PA1** and **PA4**) exhibit enhanced selectivity for their target sequences relative to cognate Nt units (i.e., **PA2**–**3**). Whilst increasing the steric bulk of the *i*Pr‐Im unit (**PA4**) does not impact DNA binding affinity for its target sequence, NMR structural analysis reveals the larger *i*Pr‐Im unit does induce more pronounced structural perturbation of the target dsDNA duplex relative to **PA1**, which contains an N‐terminal Me‐Im unit.

## Results

### Design and synthesis of hairpin polyamides (PA1–4)

The heterocyclic core of a known hairpin PA sequence (**PA1**) was chosen as our exemplar scaffold to explore the dsDNA binding profile as a function of four different N‐terminal heterocycles.4f, 5b, 8a **PA1** has an established high affinity binding profile for the general sequence 5′‐WWGWWCW (where W=A/T), for which we used 5′‐**AT*G*TACT** as the target sequence in an immobilized DNA duplex (**ODN1**).^6^, 8a, 9 Compounds **PA1**–**4** were prepared using Boc‐based solid phase synthesis on a β‐Ala PAM resin via amide coupling of the corresponding heterocyclic carboxylic acid (Scheme S1).^6^, ^10^

### Polyamides incorporating N‐terminal imidazole units exhibit picomolar binding affinity for their target dsDNA sequence

**Figure 2:**
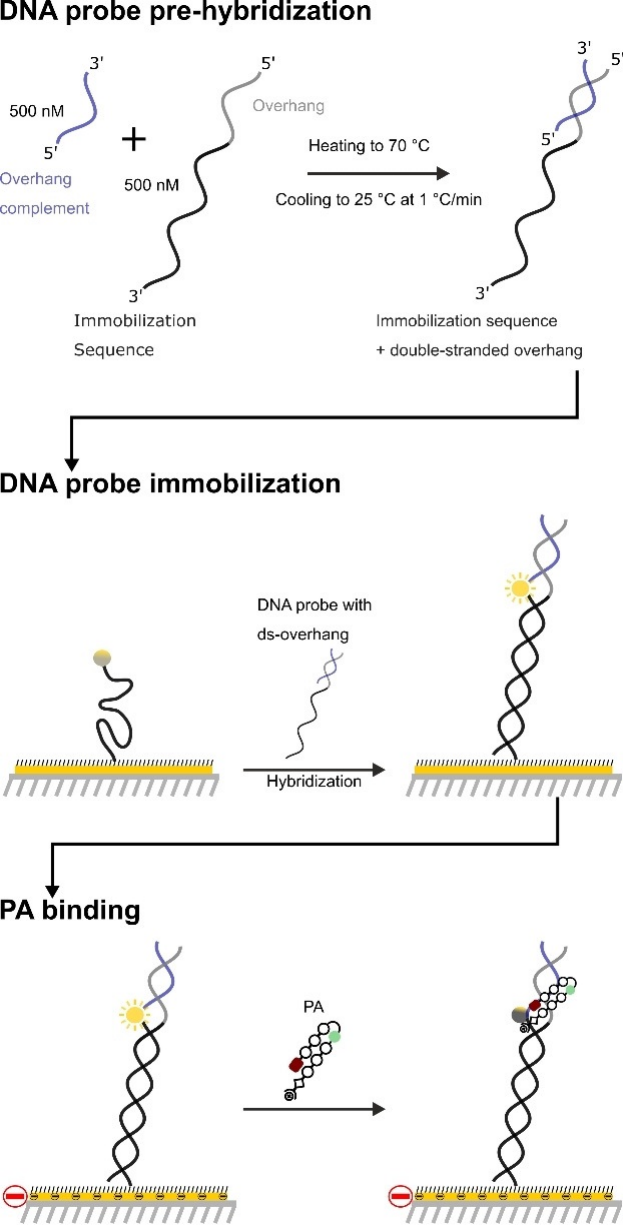
Overview of the experimental setup used to determine the binding profile of **PA1**–**4** for a suite of DNA duplexes.

A schematic of the experimental setup is shown in Figure 2. DNA duplexes (**ODN1**–**3**, Table 1) were immobilized on a gold surface and contained a fluorophore reporter positioned in close proximity to the proposed PA binding site. PA binding to an immobilized DNA duplex containing the target binding sequence (**ODN1**)^11^ results in fluorophore quenching, which is then restored upon dissociation. This provides an isothermal reporter of the binding kinetics (i.e., *k*_on_ and *k*_off_) and the equilibrium dissociation constant (*K*_D_).^12^ The same fluorescence reporter setup was also used to determine the duplex stabilization profile (i.e., Δ*T*_m_) of PA**⋅**ODN complexes as a function of a temperature gradient.

**Table 1:** Equilibrium dissociation constant (*K*_D_ [pm]) data for **PA1**–**4** binding to the target sequence (**ODN1**) versus mismatched sequences (**ODN2**–**3**).

	**PA1**	**PA2**	**PA3**	**PA4**
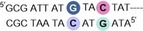	254±8	1170±70	1970±240	188±5
**ODN1**				
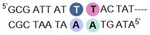	1320±70	1250±110	2880±440	967±35
**ODN2**				
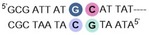	ND	15 400±7700	ND	1100±100
**ODN3**				

**Figure 3:**
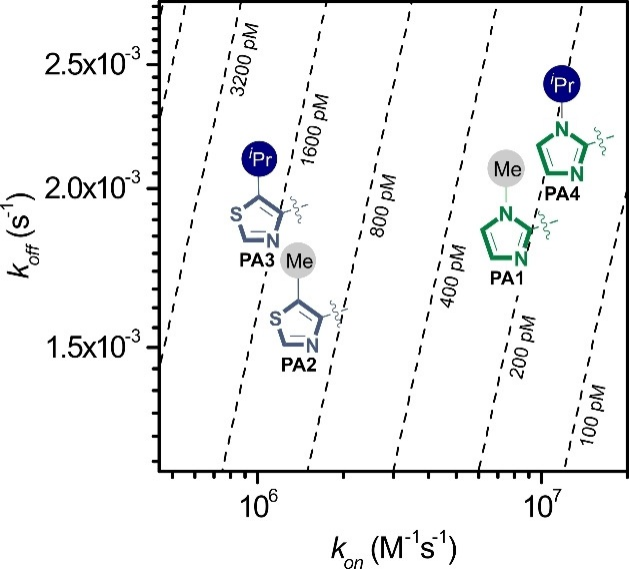
Rate maps of **PA1**–**4** binding to **ODN1**.

Kinetic analyses of the binding profile of **PA1**–**4** to **ODN1** show all four PAs exhibit high‐affinity binding (Table 1). Whilst the Im‐containing PAs (**PA1** and **PA4**) exhibit *K*_D_ values in the picomolar range, the Nt‐containing PAs (**PA2**–**3**) exhibited a binding affinity that is approximately an order of magnitude lower (i.e., in the low nanomolar range). Rate maps of **PA1**–**4** targeting **ODN1** provided deeper insight into the origin of the differences in the *K*_D_ values of our PA set (Figure 3). Although the dissociation rate (*k*_off_) for each PA was similar, the rate of association (*k*_on_) of **PA2**–**3** was approximately an order of magnitude slower relative to **PA1** and **PA4**.

### G‐selective dsDNA binding observed for all four polyamides

**Figure 4:**
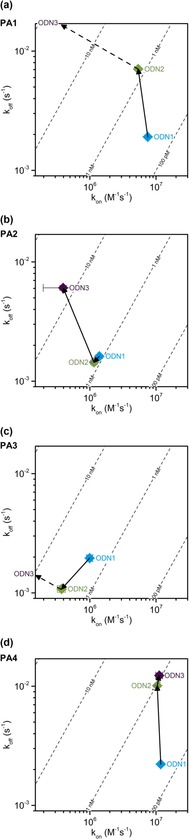
Comparative analyses of the dsDNA sequence selectivity of **PA1**–**4** binding to **ODN1**–**3**.

The sequence selectivity profile of **PA1**–**4** was explored using duplexes where the target binding sequence in **ODN1** was replaced with mismatched sequences (**ODN2**–**3**). Analyses of the binding kinetics show that Im‐containing PAs (**PA1** and **PA4**) are more G‐selective relative to Nt‐containing PAs (**PA2**–**3**, Figure 4). Whilst the rates of association (*k*_on_) of **PA4** for all sequences **ODN1**–**3** were similar, the dissociation rates (*k*_off_) were significantly faster for mismatched sequences (**ODN2**–**3**). A less pronounced *k*_on_/*k*_off_ trend was observed for **PA1** binding to **ODN2**, while no interaction was measured with **ODN3**.

Consistent with our previous DNA‐foot‐printing data,^6^ the most promiscuous dsDNA binding profile observed was **PA2** (Figure 4 b) where the *K*_D_ was virtually the same for the target (**ODN1**) and the mismatch (**ODN2**) sequence. Out of the PA series, **PA3** displayed the most unique binding profile (Figure 4 c). In this case, a decrease in both *k*_off_ and *k*_on_ was observed for the binding profile of **PA3** for **ODN2**, while no interaction was observed for **ODN3**.

This experimental setup was also used to determine duplex stabilization of PA**⋅**dsDNA complexes compared to the free DNA duplex melts. A global Boltzmann fit over three independent runs was used to determine the mid‐points of the melting transitions (*T*_m_) for free **ODN1**–**3** and in complex with 20 nm **PA1**–**4**. The UV/Vis melting profiles of the PA**⋅**dsDNA complexes confirm a similar trend in dsDNA sequence selectivity (i.e., higher Δ*T*_m_) observed in the fluorescence experiments (Figure S3). Of particular note was the melting stabilization of **PA4**, which displayed excellent G‐selectivity relative to **PA1**–**3**. Consistent with our kinetics profiling (Figure 4) and previous DNA‐foot‐printing work,^6^ **PA2** exhibited limited sequence selectivity as highlighted by duplex stabilization observed for all three ODNs. Taken collectively, the kinetic and melting analyses show that the sequence selectivity of Im‐containing PAs (i.e., **PA1** and **PA4**) is superior to Nt‐containing analogues (i.e. **PA2**–**3**). Furthermore, whilst enhancing steric bulk on the 5‐position of the Nt‐series enhanced G‐selectivity, this had little effect on the Im‐series (i.e., **PA1**/**PA4**).

### NMR structural analysis of the PA4⋅dsDNA complex

**Figure 5:**
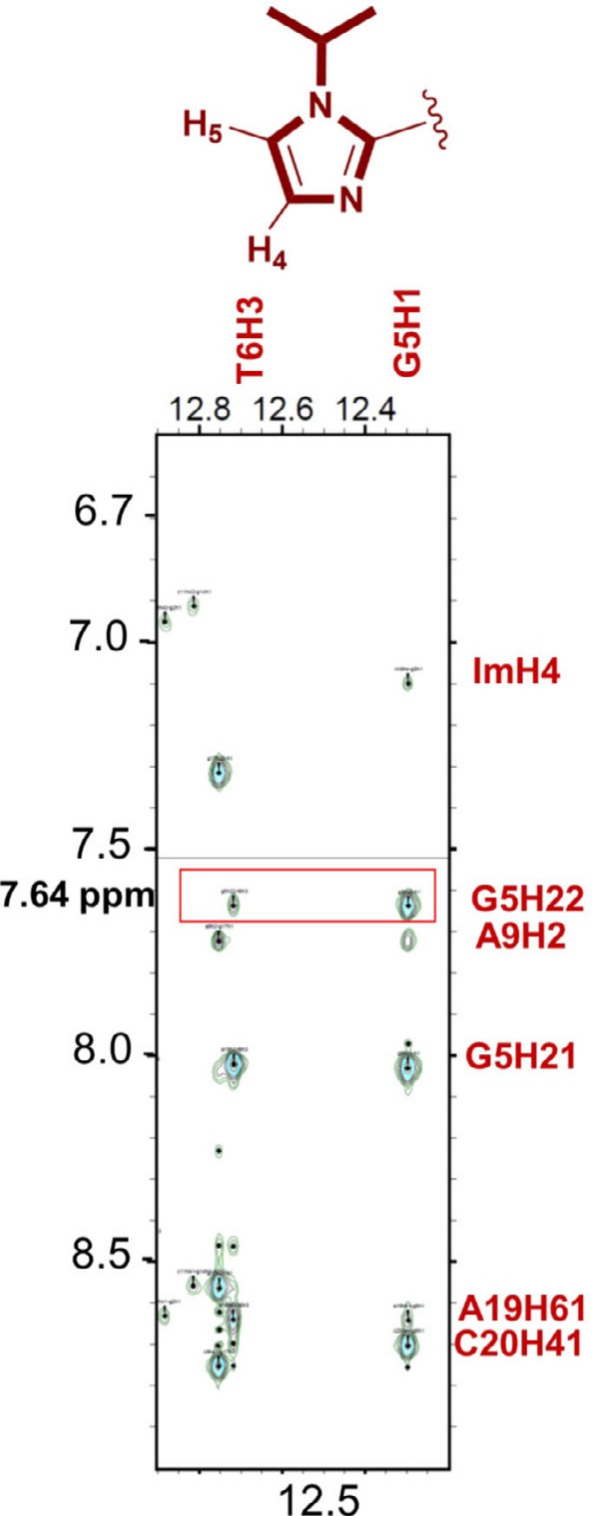
Strip plot analysis of 2D [^1^H, ^1^H] NOESY NMR data of **PA4⋅ODN4**.

In order to gain insight into the influence of the *i*Pr‐Im unit incorporated in **PA4** when in complex with its target dsDNA sequence, NMR studies were undertaken using the self‐complementary dodecamer sequence d(CG**AT*G*TACA**TCG)_2_ (**ODN4**). Titration experiments of **PA4** into a solution of **ODN4** confirmed the formation of a 1:1 **PA4⋅ODN4** complex. 2D NOESY studies at 4 different mixing times identified a suite of strong NOE cross‐correlations from H4 of the *i*Pr‐Im building block to G5H1 and the G5N2 exocyclic amine, which implies that the *i*Pr‐Im N3 is directed towards the floor of the minor groove (Figure 5; Figure S9). NOE cross‐peaks from H4 and H5 of the *i*Pr‐Im building block to Py1 and the β‐alanine tail in the **PA4⋅ODN4** complex is indicative of the PA binding to its target sequence in the hairpin conformation.

### Comparative NMR structural analyses of polyamide⋅dsDNA complexes

**Figure 6:**
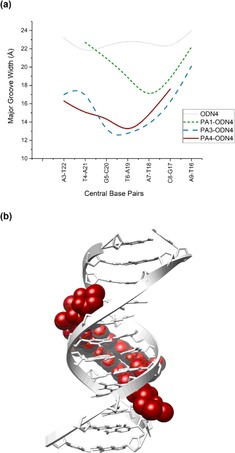
(a) Major groove width of **ODN4** (grey), **PA1⋅ODN4** (green), **PA3⋅ODN4** (blue), and **PA4⋅ODN4** (red). NMR‐derived molecular model of (b) the **PA4⋅ODN4** complex.

Previous NMR structural work highlighted an increased propensity of **PA3** to compress the major groove when in complex with its target DNA sequence (**PA3⋅ODN4**) *relative* to **PA1⋅ODN4**. A similar trend in enhanced major groove compression was also observed with **PA4⋅ODN4** relative to **PA1⋅ODN4** (Figure 6). However, the extent of major groove compression was not as pronounced as that observed for the **PA3⋅ODN4** complex.

**Figure 7:**
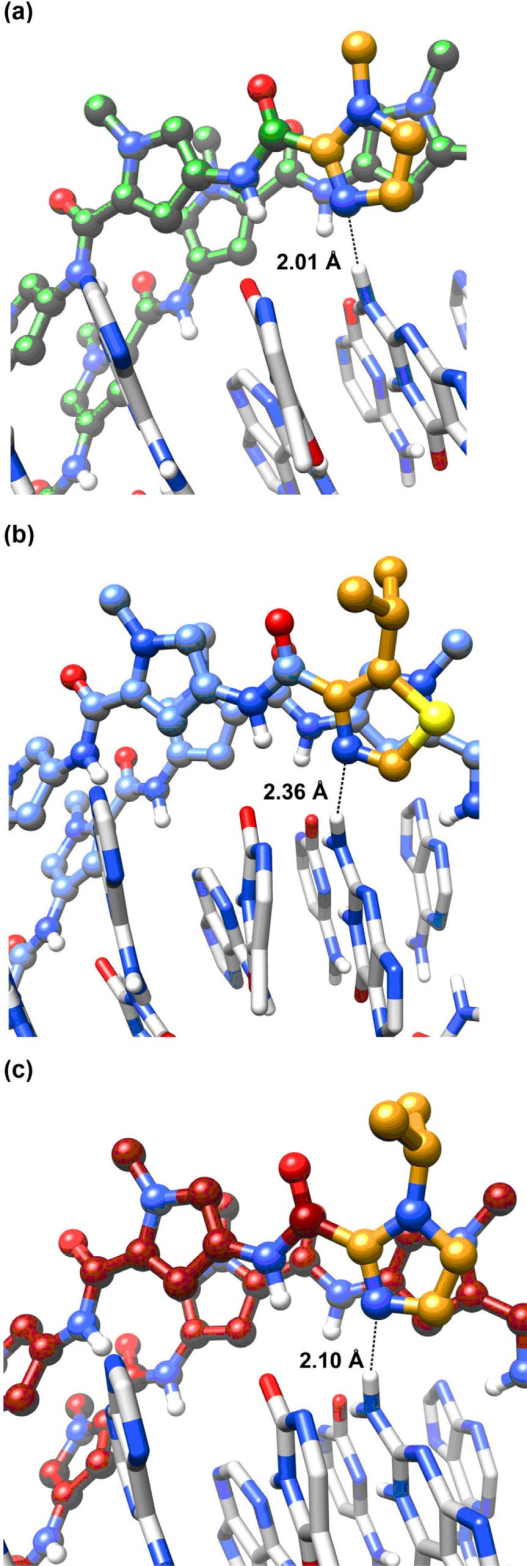
Comparative analysis of the minor groove penetration of (a) **PA1⋅ODN4**, **PA3⋅ODN4**, and **PA4⋅ODN4** (**PA4⋅ODN4** structure produced from average of ensemble of clusters from last 800 ps of 1 ns MD simulations; **PA1⋅ODN4** and **PA3⋅ODN4** structures produced through Chimera from averaged clusters from PDB deposition IDs 5OE1 and 5ODM, respectively).

The origins of these differences become apparent when comparing the extent of minor groove penetration of the three complexes (Figure 7). NMR‐restrained molecular dynamics of the **PA1⋅ODN4** complex reveal **PA1** penetrating deep within the minor groove, exemplified by a hydrogen bond distance of 2.01 Å between Me‐Im N3 and the exocyclic amine G5N2 (Figure 7 a).^13^ In contrast, the **PA3⋅ODN4** complex shows a reduced level of minor groove penetration with an average distance of 2.36 Å between the *i*Pr‐Nt N3 and the exocyclic amine G5N2 (Figure 7 b).^13^ The **PA4⋅ODN4** complex on the other hand shows a significant level of minor groove penetration (2.10 Å) relative to **PA3⋅ODN4** but it is not as extensive as that observed for the **PA1⋅ODN4** complex (2.01 Å).^6^ We therefore conclude that both the nature of the N‐terminal heterocycle and the steric bulk distal to the DNA‐binding face of a PA scaffold influences the allosteric modulation of a target dsDNA sequence.

## Discussion

This combined kinetic and structural study has shown that the type of N‐terminal heterocycle and its substituents influences the dsDNA binding profile and the overall structure of the duplex. We discuss here several conclusions that emerged from our results.

### N‐terminal heterocycle of a hairpin polyamide influences rate of association to target dsDNA sequence

Firstly, all four PAs exhibit high affinity (low nanomolar‐picomolar) for its target dsDNA sequence. However, the two N‐terminal Im‐containing PAs (**PA1**/**PA4**) showed a higher binding affinity relative to the Nt‐containing **PA2**–**3** via an increase in the rate of association. Although there has not been a study dedicated to evaluating the influence of the hairpin PA N‐terminus, a previous SPR‐based study by Sugiyama et al. has shown that the number of Me‐Im and their positioning in a hairpin PA scaffold can have a disproportionate impact on the *k_a_* and *K_D_* relative to only small changes in the *k*_d._ 14 In contrast, replacing internal Py/Im heterocycles with more flexible *β*‐alanine units influences both *k_a_* and *k_d_* parameters.^15^ Extensive work by Dervan et al. has investigated heterocyclic changes to the internal positions of hairpin PA structures.^16^ However, our results highlight the N‐terminal position can be used as a convenient site to tune parameters of dsDNA binding and overall physicochemical properties.

### The N‐terminal heterocycle position of hairpin polyamides influence DNA structural perturbations

Our structural and binding analyses show that whilst an increase in the steric bulk of the *i*Pr‐Im unit does not impact dsDNA binding affinity to its target binding site (i.e., **PA4⋅ODN4** complex), an improvement in G‐selectivity *relative* to the *i*Pr‐Nt unit (i.e., **PA3⋅ODN4** complex) is likely due to a greater level of minor groove penetration (see Figure 7), and in turn improved recognition of the N2 amine of G. However, the extent of major groove compression of the **PA4⋅ODN4** complex (Figure 6a) is less than in **PA3⋅ODN4** (Figure 7). This suggests a fine interplay between minor groove penetration versus major groove compression, with enhanced major groove compression occurring if the hydrogen‐bond between the N‐terminal building block and the N2 of G is weaker as in **PA3⋅ODN4**, thereby reducing penetration of the minor groove.

## Conclusions

These experiments were designed to probe how an increase in the steric bulk of heterocyclic building blocks of PA impacts the binding kinetics and the allosteric distortion of dsDNA containing the target binding sequence. Although what superficially appears to be a subtle increase in steric bulk at locations within a PA scaffold not directly involved in dsDNA base‐readout, these data suggest that strategic changes in the Im and Nt substitution pattern can be used to fine tune the sequence‐selectivity of dsDNA binding as well as the overall physicochemical properties of PA scaffolds.^17^ We envisage that the strategic incorporation of modified heterocyclic building blocks within a PA scaffold could be applied more broadly as a strategy to enhance cell uptake and potency of transcriptional modulation in cellulo.

## Conflict of interest

The authors declare no conflict of interest.

## Supporting information

As a service to our authors and readers, this journal provides supporting information supplied by the authors. Such materials are peer reviewed and may be re‐organized for online delivery, but are not copy‐edited or typeset. Technical support issues arising from supporting information (other than missing files) should be addressed to the authors.

Supplementary
