# Supplementary material for: Structural and Kinetic Profiling of Allosteric Modulation of Duplex DNA Induced by DNA‐Binding Polyamide Analogues
Source: Chemistry. 2019 Jan 14;25(11):2757–63. doi: 10.1002/chem.201805338 (PMC6468288; doi:10.1002/chem.201805338)
Supplement: Supplementary file 1 — Supplementary [file CHEM-25-2757-s001.pdf]

CHEMISTRY
A European Journal

Supporting Information

Structural and Kinetic Profiling of Allosteric Modulation of Duplex DNA Induced by DNA-Binding Polyamide Analogues

Khalid Aman+,[a] Giacomo Padroni+,[a] John A. Parkinson,[a] Thomas Welte,*[b] and Glenn A. Burley*[a]

chem_201805338_sm_miscellaneous_information.pdf

Structural and Kinetic Profiling of Allosteric Modulation of Duplex DNA Induced by DNA-Binding Polyamide Analogues

Khalid Aman[a]‡, Giacomo Padroni[a]‡, John A. Parkinson[a], Thomas Welte[b]* and Glenn A. Burley[a]*

SUPPORTING INFORMATION

Contents

1.0 Supplementary Figures

Figures S1 to S10 2-13

2.0 Supplementary Tables

Tables S1 to S15 13-27

3.0 Supplementary Methods 28-46

3.1 General Procedures 28

3.2 Synthesis of PA4 33

3.3 Dynamic Biosensors switchSENSE® methods 39

3.4 NMR methods 41

4.0 References 45-46

[a] Mr Khalid Aman (K.A.), Dr Giacomo Padroni (G.P.), Dr John A. Parkinson (J.A.P.) and Professor Glenn A. Burley (G.A.B.)
Department of Pure and Applied Chemistry
University of Strathclyde
295 Cathedral Street, Glasgow, United Kingdom, G1 1XL
E-mail: glenn.burley@strath.ac.uk

‡ Authors contributed equally.

[b] Mr Thomas Welte (T.W.)
Dynamic Biosensors GmbH,
82152 Planegg, Germany
E-mail: welte@dynamic-biosensors.com

1.0 Supplementary Figures

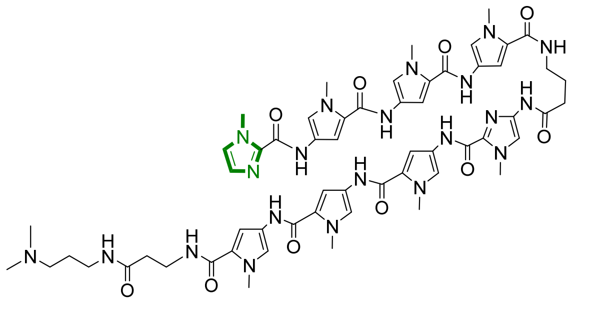

PA1

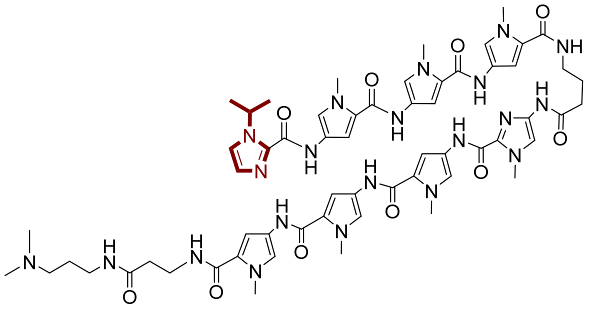

PA4

Figure S1. Structures of **PA1** and **PA4**

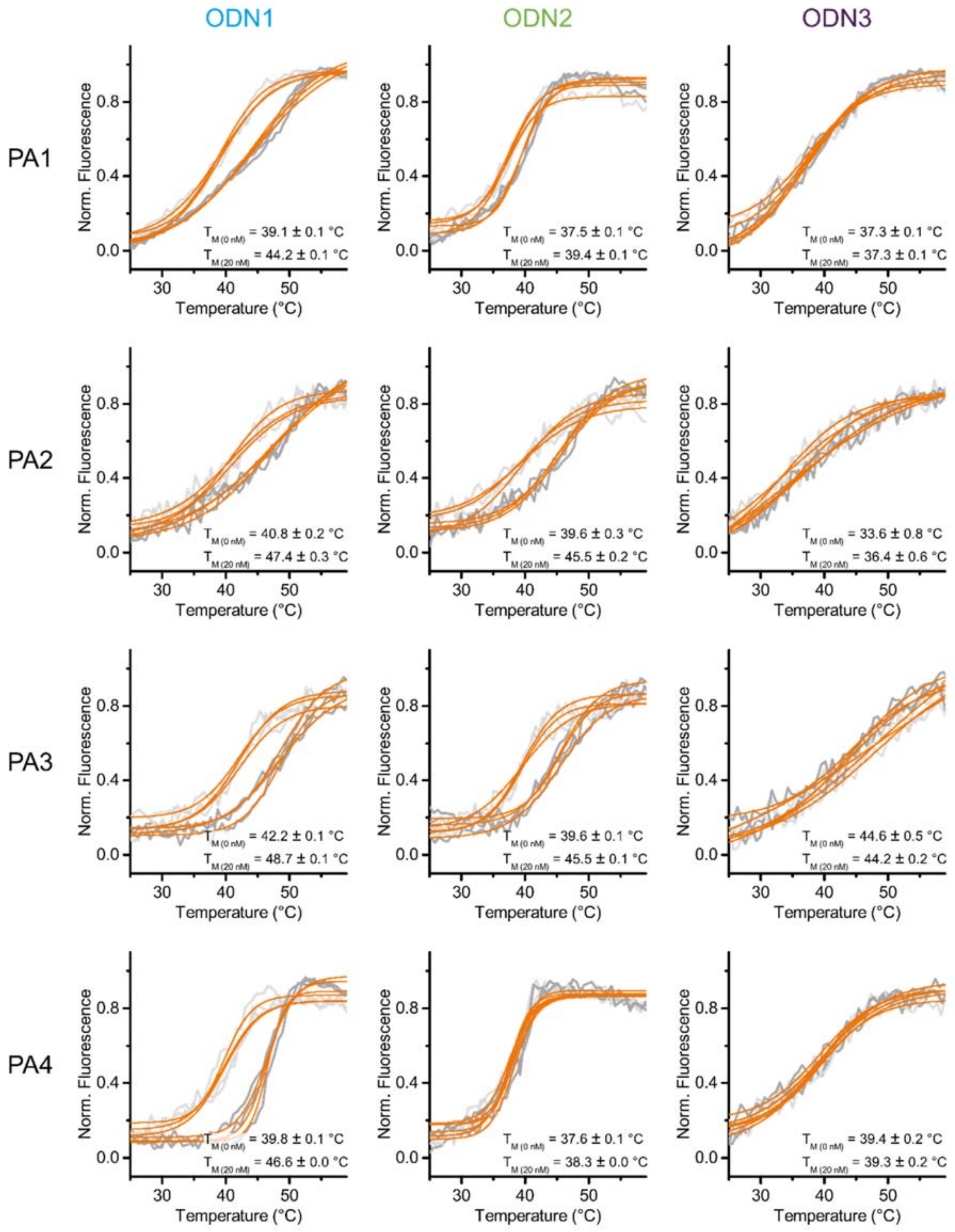

Figure S2. Fluorescence melting curves of **PA1-4** for **ODN1-3** ($\Delta T_m = T_m$complex - T_mODN1-3; error bars were calculated from 3 independent runs each with 0 nM and 20 nM PA1-4).

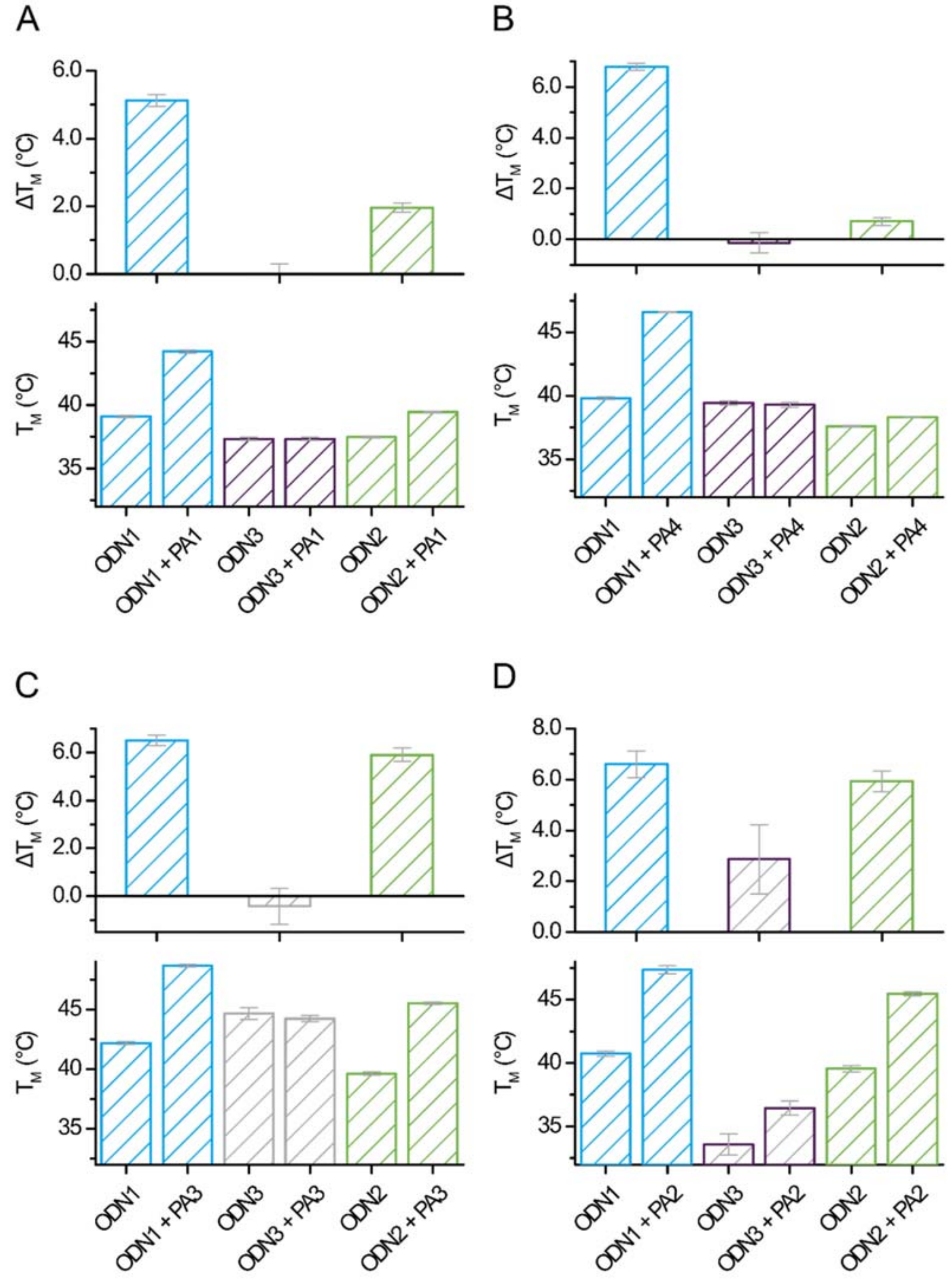

Figure S3. Fluorescence melting temperature comparisons of **PA1-4** with **ODN1-3**.

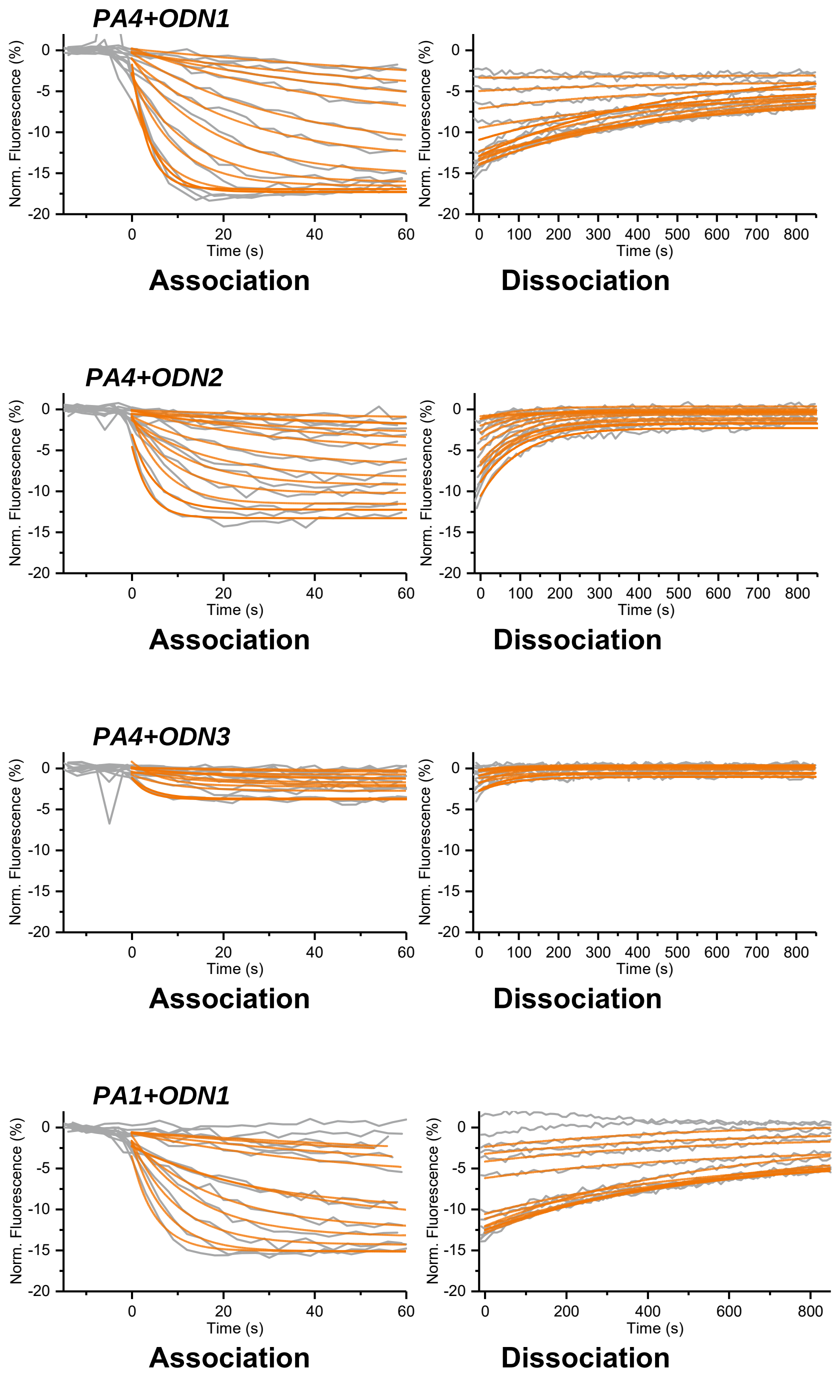
PA4+ODN1
PA4+ODN2
PA4+ODN3
PA1+ODN1
Norm. Fluorescence (%)
Time (s)
Association
Dissociation

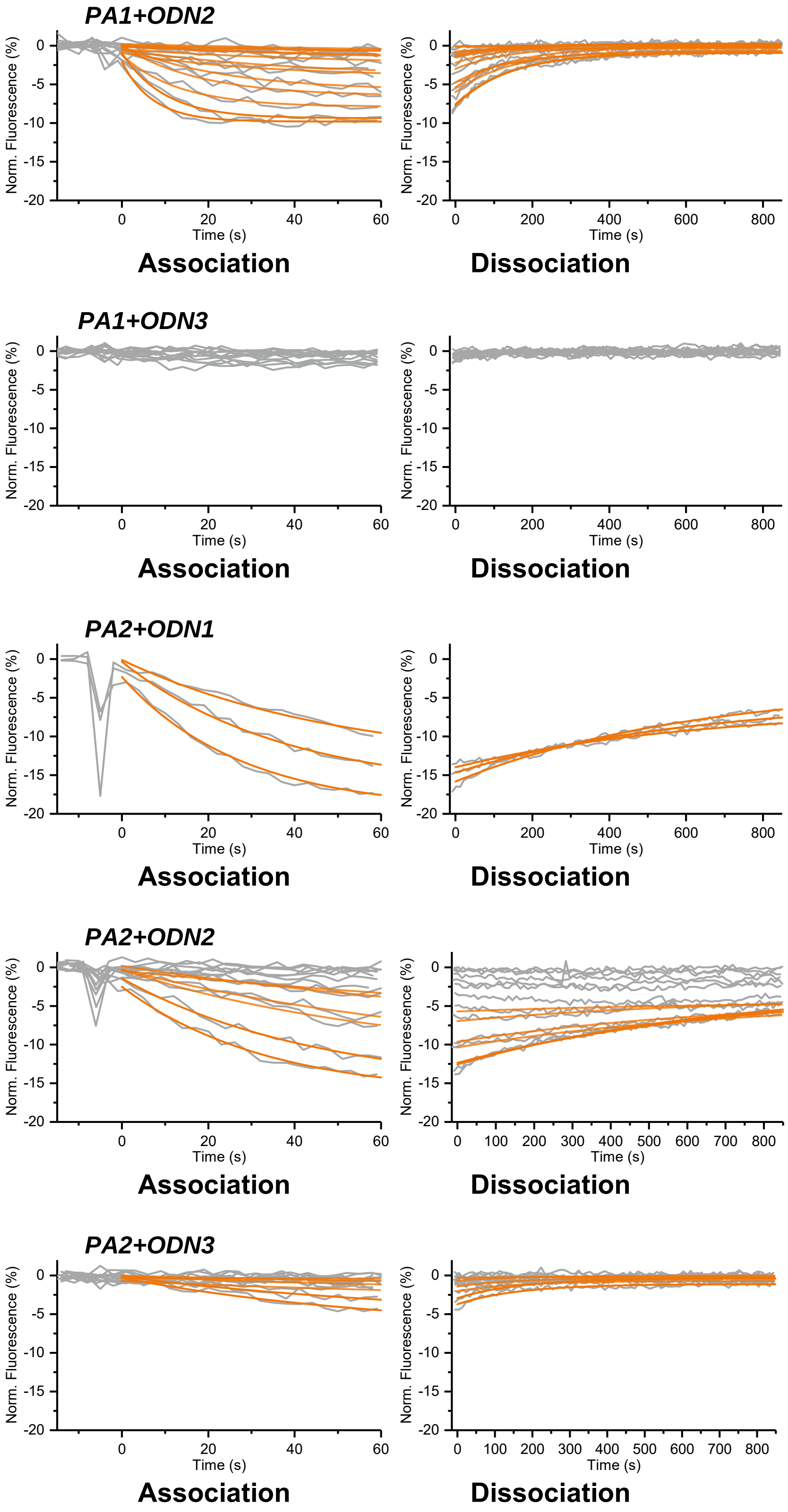
PA1+ODN2
PA1+ODN3
PA2+ODN1
PA2+ODN2
PA2+ODN3
Norm. Fluorescence (%)
Time (s)
Association
Dissociation

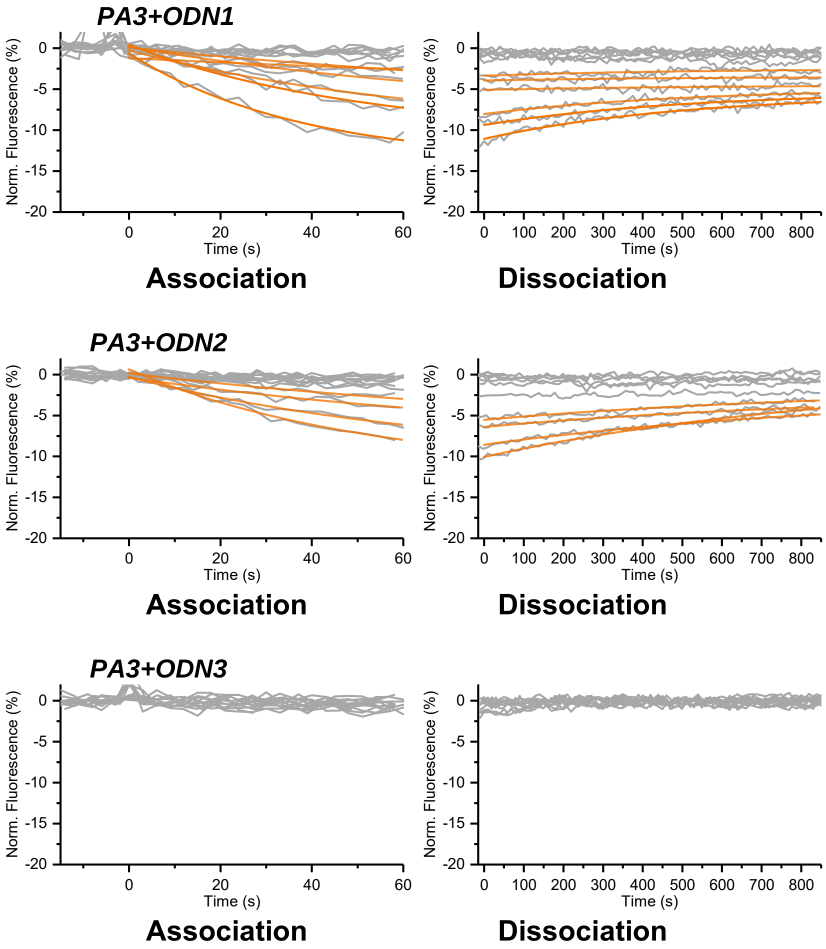

Figure S4. Association and dissociation fluorescence response curves for **PA1-4** with **ODN1-3** at PA concentrations ranging from 450 pM to 25.5 nM (up to 49 nM in **PA3-ODN2** case)

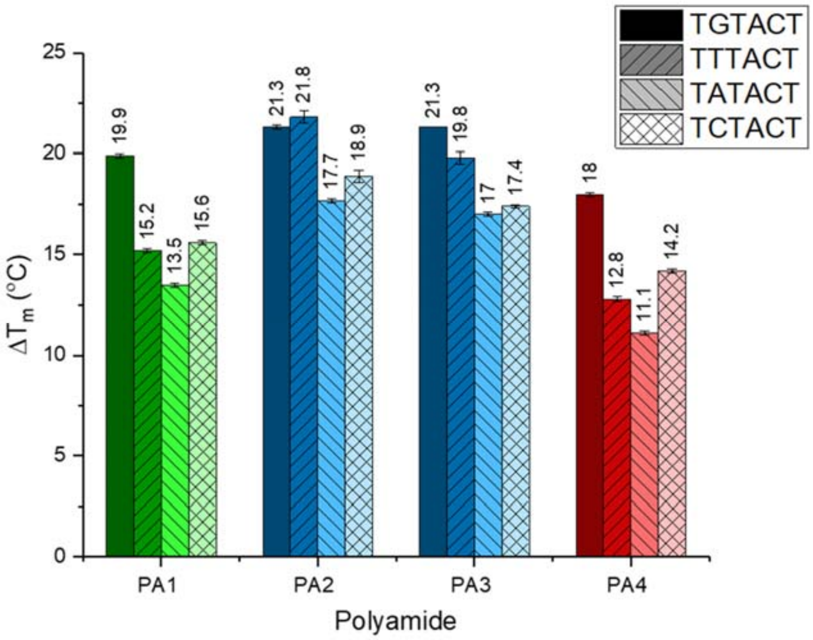

Figure S5. DNA UV melt stabilization of **PA1-4** for target dsDNA sequence and single-point mutations (sequence: 5'CG*ATXTACA*TGC where X = G/C/A/T; error bars calculated from the standard deviation of three independent measurements. Underlined sequence corresponds to the PA binding site. PA1, PA2, PA3 data are reproduced here from G. Padroni in NAR[1] for comparison with PA4).

(a)

(b)

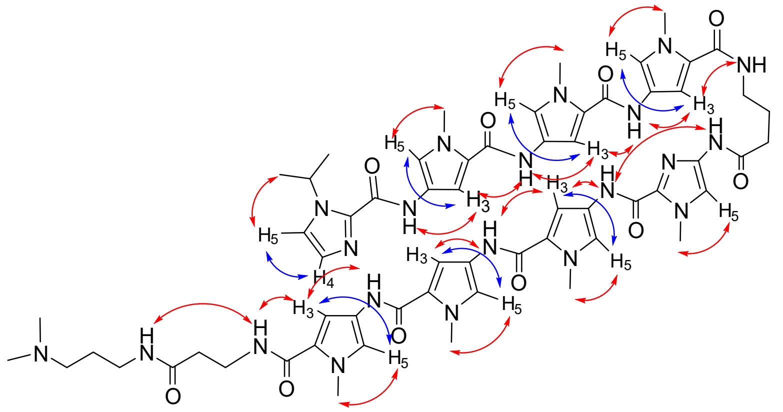

Figure S6. (a) Schematic showing the inter-residue hydrogen bonds observed for PA4•ODN4; (b) ^{1}H NMR 2D NOESY and TOCSY correlations used for **PA4** proton assignment. Red arrows indicate observed NOESY cross correlations; blue arrows indicate both NOESY and TOCSY observed correlations.

(a)

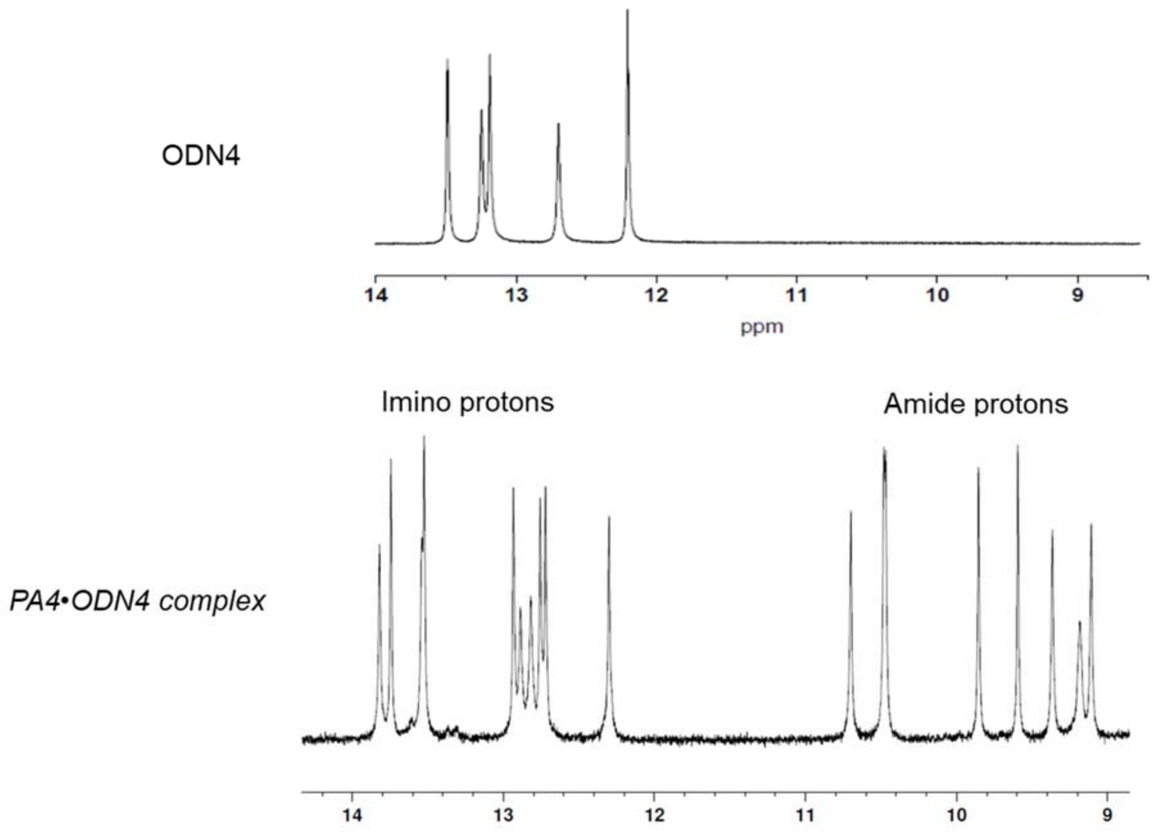

(b)

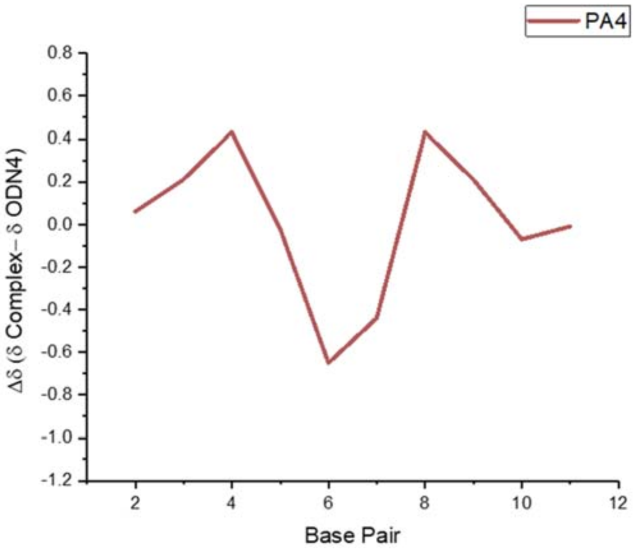
PA4
Δδ (δ Complex– δ ODN4)
Base Pair
0.8
0.6
0.4
0.2
0.0
-0.2
-0.4
-0.6
-0.8
-1.0
-1.2
2
4
6
8
10
12

(c)

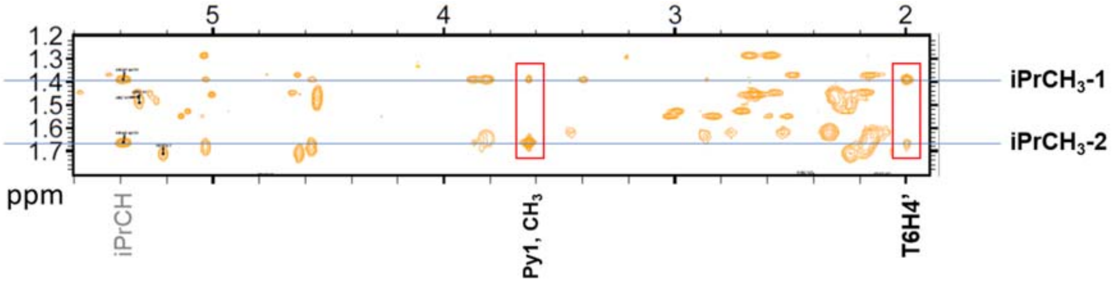
5
4
3
2
1.2
1.3
1.4
1.5
1.6
1.7
ppm
iPrCH₃-1
iPrCH₃-2
iPrCH
Py1, CH₃
T6H4'

(d)

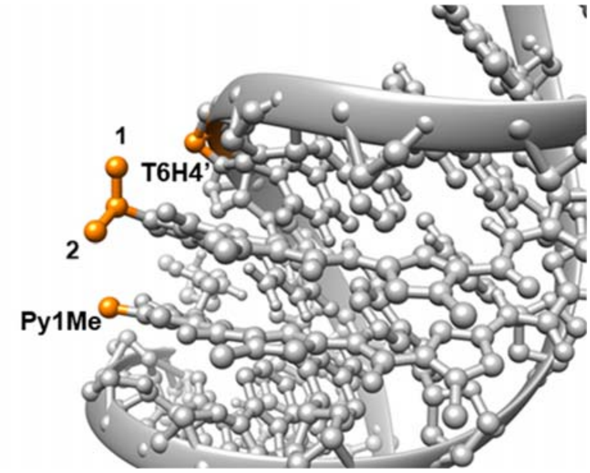

Figure S7. (a) 1D ^{1}H-NMR imino-amide chemical shift region of free **ODN4** and **PA4•ODN4** complex (b) chemical shift perturbation plot of imino resonances (c) 2D ^{1}H-^{1}H NOESY spectrum strip plot of **PA4•ODN4** complex highlighting key NOE contacts for determining the orientation of the isopropyl group in the minor groove (d) orientation of the isopropyl group in the DNA minor groove highlighted in orange (average structure from the clustering of the last 800 ps of the 1 ns molecular dynamics simulation)

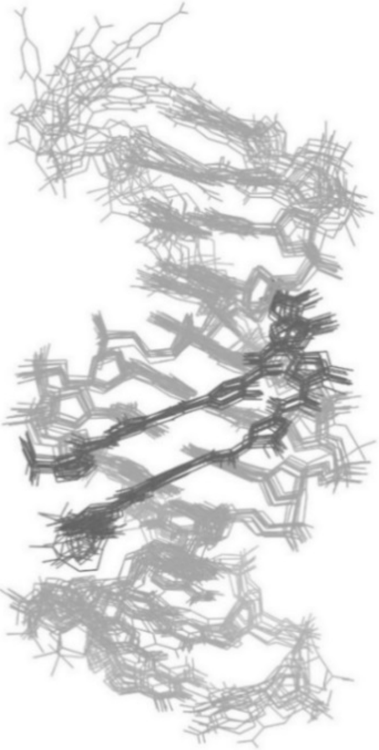

Figure S8. Ensemble of conformations of **PA4•ODN4** complex obtained from clustering of the last 800 ps of the 1 ns molecular dynamics production run.

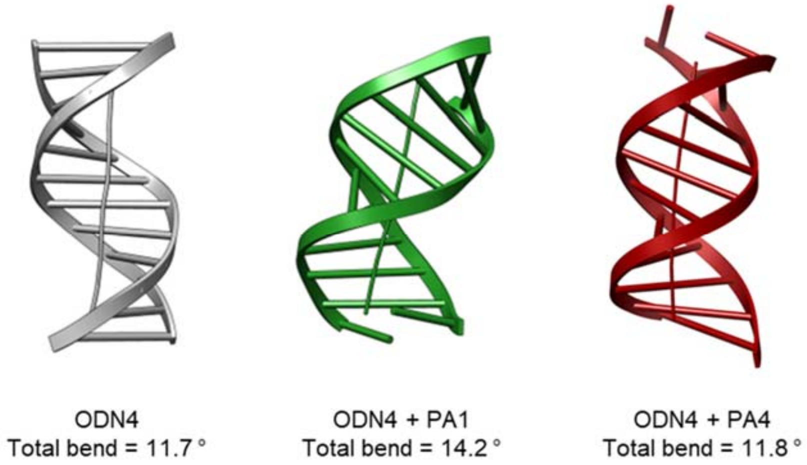

Figure S9. Axis bending imparted on the dsDNA duplex (**ODN4**) upon binding of **PA1** and **PA4** (polyamide removed for clarity).

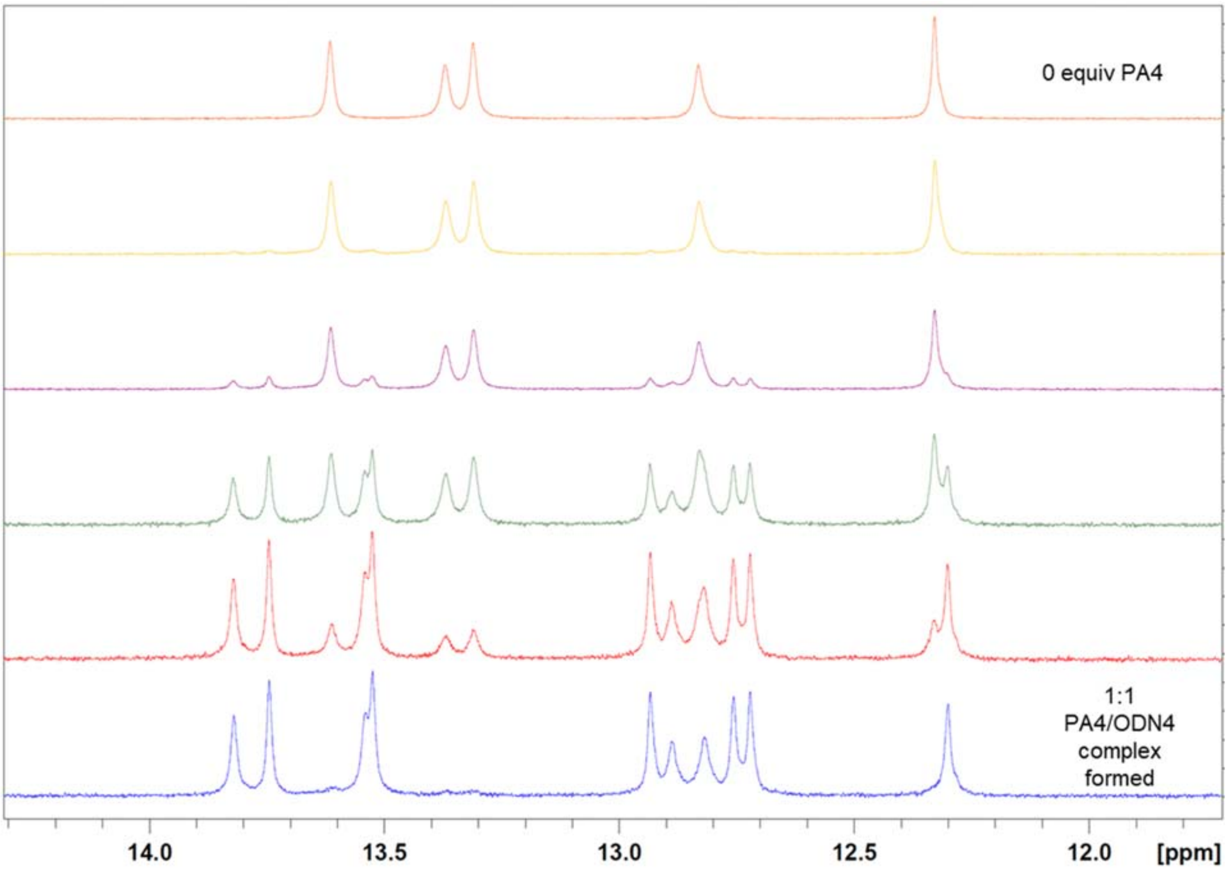

Figure S10. NMR titration of **PA4** aqueous solution (15 mM) into **ODN4** (1.5 mM solution in 90% H_2O/10% D_2O, with pH 7.4 phosphate buffer).

2.0 Supplementary Tables

Table S1. DNA sequences immobilized for switchSENSE® kinetics / T_m experiments.

DNA sequences studied	
ODN1	5'-**GCGATT** **ATGTACT** ATC AGC GTT CGA TGC TTC CGA CTA ATC AGC CAT ATC AGC TTA CGA CTA-3'
ODN2	5'-**GCGATT** **ATTTACT** ATC AGC GTT CGA TGC TTC CGA CTA ATC AGC CAT ATC AGC TTA CGA CTA-3'
ODN3	5'-**GCGATT** **ATGCATT** ATC AGC GTT CGA TGC TTC CGA CTA ATC AGC CAT ATC AGC TTA CGA CTA-3'

Target Sequence

Stabilization sequence

Immobilization sequence

Table S2. K_D and rate constants from switchSENSE® experiments, along with melt temperatures recorded.

Sample	k_{ON} ($M^{-1}s^{-1}$)	Error k_{ON} ($M^{-1}s^{-1}$)	k_{OFF} (s^{-1})	Error k_{OFF} (s^{-1})	K_D (pM)	Error K_D (pM)	ΔT_m at 20 nM (°C)	Error ΔT_m (°C)
PA1 + ODN1	7.49×10^6	2.10×10^5	1.91×10^{-3}	3.00×10^{-5}	254	8	5.12	0.17
PA1 + ODN2	5.37×10^6	2.80×10^5	7.07×10^{-3}	1.00×10^{-4}	1320	70	1.97	0.13
PA1 + ODN3	No interaction	No interaction	No interaction	No interaction	No interaction	No interaction	0.00	0.29

Sample	k_{ON} ($M^{-1}s^{-1}$)	Error k_{ON} ($M^{-1}s^{-1}$)	k_{OFF} (s^{-1})	Error k_{OFF} (s^{-1})	K_D (pM)	Error K_D (pM)	ΔT_m at 20 nM (°C)	Error ΔT_m (°C)
PA2 + ODN1	1.39×10^6	8.00×10^4	1.62×10^{-3}	5.00×10^{-5}	1170	70	6.60	0.53
PA2 + ODN2	1.15×10^6	9.00×10^4	1.44×10^{-3}	6.00×10^{-5}	1250	110	5.93	0.40
PA2 + ODN3	3.93×10^5	1.96×10^5	6.07×10^{-3}	2.70×10^{-4}	15400	7700	2.87	1.37

Sample	k_{ON} ($M^{-1}s^{-1}$)	Error k_{ON} ($M^{-1}s^{-1}$)	k_{OFF} (s^{-1})	Error k_{OFF} (s^{-1})	K_D (pM)	Error K_D (pM)	ΔT_m at 20 nM (°C)	Error ΔT_m (°C)
PA3 + ODN1	9.92×10^5	1.01×10^5	1.96×10^{-3}	1.20×10^{-4}	1970	240	6.50	0.22
PA3 + ODN2*	3.74×10^5	5.20×10^4	1.08×10^{-3}	7.00×10^{-5}	2880	440	5.81	0.23
PA3 + ODN3	No Interaction	No Interaction	No Interaction	No Interaction	No Interaction	No Interaction	-0.41	0.75

Sample	k_{ON} ($M^{-1}s^{-1}$)	Error k_{ON} ($M^{-1}s^{-1}$)	k_{OFF} (s^{-1})	Error k_{OFF} (s^{-1})	K_D (pM)	Error K_D (pM)	ΔT_m at 20 nM (°C)	Error ΔT_m (°C)
PA4 + ODN1	1.18×10^7	3.00×10^5	2.22×10^{-3}	3.00×10^{-5}	188	5	6.78	0.14
PA4 + ODN2	1.04×10^7	4.00×10^5	1.01×10^{-2}	1.00×10^{-4}	967	35	0.71	0.08
PA4 + ODN3	1.12×10^7	1.30×10^6	1.23×10^{-2}	6.00×10^{-4}	1100	100	-0.13	0.39

* For PA3 + ODN2, an extended concentration regime was employed (up to 49 nM) as, despite evident association of PA3 to ODN2, the global exponential fit did not converge for the concentration regime used in the standard protocol

UV Melt data

Table S3. dsDNA T_m analysis of free DNA with and without **PA1**, **PA4** for target sequence as well as single point mutations (shown is average value from 3 melting measurements).

	5'-CGAT**GTAC**TAGC-3'		5'-CGAT**TTAC**TAGC-3'	
	T_m (°C)	ΔT_m (°C)	T_m (°C)	ΔT_m (°C)
Free DNA	46.7 ± 0.1	-	44.0 ± 0.1	-
DNA + PA4	64.6 ± 0.1	18.0 ± 0.1	56.8 ± 0.1	12.8 ± 0.1

	5'-CGAT**CTAC**TAGC-3'		5'-CGAT**ATAC**TAGC-3'	
	T_m (°C)	ΔT_m (°C)	T_m (°C)	ΔT_m (°C)
Free DNA	45.3 ± 0.1	-	41.5 ± 0.1	-
DNA + PA4	59.6 ± 0.1	14.2 ± 0.1	52.7 ± 0.1	11.1 ± 0.1

Table S4. Statistics and output of NMR restraints used for MD model determination of **PA4•ODN4**.

NMR	Total Restraints Applied	DNA	Polyamide	Inter-residue	Rmsd* (bond)	Rmsd* (angle)	Penalty* (Kcal)
**PA4**	310	181	77	52	0.0073	4.439	280.86

*output values from the last frame of 1 ns MD simulation

Table S5. ^{1}H nucleotide base chemical shifts of **PA4•ODN4** complex.

**ODN4**	**H1**	**H2**	**H21**	**H22**	**H3**	**H41**	**H42**	**H5**	**H6**	**H61**	**H62**	**H7**	**H8**
**C1**	-	-	-	-	-	n.d.	n.d.	5.90	7.60	-	-	-	-
**G2**	12.88	-	-	-	-	-	-	-	-	-	-	-	8.03
**A3**	-	7.98	-	-	-	-	-	-	-	n.d.	n.d.	-	8.45
**T4**	-	-	-	-	13.74	-	-	-	7.43	-	-	1.55	-
**G5**	12.30	-	8.03	7.64	-	-	-	-	-	-	-	-	8.00
**T6**	-	-	-	-	12.72	-	-	-	6.97	-	-	1.29	-
**A7**	-	8.05	-	-	-	-	-	-	-	8.46	5.59	-	8.14
**C8**	-	-	-	-	-	8.75	6.43	5.27	6.88	-	-	-	-
**A9**	-	7.72	-	-	-	-	-	-	-	n.d.	n.d.	-	8.30
**T10**	-	-	-	-	13.54	-	-	-	6.97	-	-	1.45	-
**C11**	-	-	-	-	-	8.56	6.91	5.62	7.42	-	-	-	-
**G12**	n.d.	-	n.d.	n.d.	-	-	-	-	-	-	-	-	7.94
**C13**	-	-	-	-	-	n.d.	n.d.	5.93	7.66	-	-	-	-
**G14**	12.81	-	n.d.	n.d.	-	-	-	-	-	-	-	-	8.01
**A15**	-	7.90	-	-	-	-	-	-	-	n.d.	n.d.	-	8.33
**T16**	-	-	-	-	13.52	-	-	-	7.28	-	-	1.53	-
**G17**	12.75	-	8.56	7.32	-	-	-	-	-	-	-	-	8.02

**T18**	-	-	-	-	12.93	-	-	-	6.98	-	-	1.45	-
**A19**	-	8.03	-	-	-	-	-	-	-	8.64	5.57	-	8.15
**C20**	-	-	-	-	-	8.70	6.43	5.25	6.85	-	-	-	-
**A21**	-	7.73	-	-	-	-	-	-	-	n.d.	n.d.	-	8.27
**T22**	-	-	-	-	13.82	-	-	-	6.96	-	-	1.37	-
**C23**	-	-	-	-	-	8.63	6.95	5.66	7.47	-	-	-	-
**G24**	n.d.	-	n.d.	n.d.	-	-	-	-	-	-	-	-	7.95

Table S6. ^{1}H sugar backbone chemical shifts of **PA4•ODN4** complex.

ODN4	H1'	H2'	H2''	H3'	H4'	H5'	H5''
**C1**	5.790	1.886	2.385	4.717	4.077	3.708	3.743
**G2**	5.460	2.869	2.777	5.043	4.329	n.d.	n.d.
**A3**	6.367	2.834	2.834	5.137	4.525	n.d.	n.d.
**T4**	5.775	2.518	2.590	5.047	4.275	n.d.	n.d.
**G5**	6.020	2.587	2.674	5.035	4.243	n.d.	n.d.
**T6**	5.032	1.683	2.174	4.572	1.995	n.d.	n.d.
**A7**	5.533	2.213	2.701	4.673	2.863	n.d.	n.d.
**C8**	5.325	1.457	2.311	4.547	2.223	n.d.	n.d.
**A9**	5.574	2.175	2.571	4.663	2.675	n.d.	n.d.
**T10**	5.699	1.869	2.306	4.765	3.917	n.d.	n.d.
**C11**	5.748	1.970	2.369	4.824	4.116	n.d.	n.d.
**G12**	6.160	2.369	2.601	4.681	4.181	n.d.	n.d.
**C13**	5.767	1.988	2.441	4.732	4.077	n.d.	n.d.
**G14**	5.678	2.775	2.893	5.049	4.372	n.d.	n.d.
**A15**	6.335	2.712	2.993	5.109	4.512	n.d.	n.d.
**T16**	5.636	2.259	2.385	4.934	4.148	n.d.	n.d.
**G17**	5.796	2.654	2.654	5.005	4.273	n.d.	n.d.
**T18**	5.215	1.711	2.242	4.627	2.062	n.d.	n.d.
**A19**	5.526	2.191	2.712	4.667	2.645	n.d.	n.d.
**C20**	5.319	1.486	2.294	4.549	2.297	n.d.	n.d.
**A21**	5.446	2.157	2.488	4.634	2.682	n.d.	n.d.
**T22**	5.745	1.837	2.103	4.771	3.785	n.d.	n.d.
**C23**	5.510	2.095	2.318	4.781	4.117	n.d.	n.d.
**G24**	6.189	2.403	2.629	4.684	4.171	n.d.	n.d.

Table S7. ^{1}H **PA4** chemical shifts of **PA4•ODN4** complex (' and ' ' refer to the most upfield and the most downfield signal, respectively).

PA4	H2'	H2"	H3	H3'	H3"	H4	H4'	H4"	H5	CH_3	NH	iPr1*Me*	iPr2*Me*	iPrC*H*
**Dp**	3.14	3.18	-	1.95	2.13	-	2.97	3.63	-	-	8.08	-	-	-
**β-Ala**	1.63	2.15	-	2.88	3.82	-	-	-	-	-	9.18	-	-	-
**Py1**	-	-	6.30	-	-	-	-	-	7.07	3.63	9.36	-	-	-
**Py2**	-	-	6.35	-	-	-	-	-	7.50	3.72	10.70	-	-	-
**Py3**	-	-	6.20	-	-	-	-	-	7.60	3.87	9.85	-	-	-
**Im4**	-	-	-	-	-	-	-	-	7.46	3.95	10.48	-	-	-
**Turn**	2.10	2.53	-	1.62	2.33	-	2.75	3.44	-	-	8.24	-	-	-
**Py5**	-	-	6.46	-	-	-	-	-	7.10	3.59	9.11	-	-	-
**Py6**	-	-	6.24	-	-	-	-	-	7.62	3.79	10.47	-	-	-
**Py7**	-	-	6.05	-	-	-	-	-	7.58	3.91	9.59	-	-	-
**Im8**	-	-	-	-	-	7.10	-	-	7.28	-	-	1.39	1.66	5.39

PA4	C3	C4	C5	*N*-CH3	iPr1*Me*	iPr2*Me*	iPr*C*H
**Py1**	106.8	-	119.4	36.6	-	-	-
**Py2**	102.5	-	121.8	37.1	-	-	-
**Py3**	104.4	-	-	37.8	-	-	-
**Im4**	-	-	114.1	36.4	-	-	-
**Py5**	105.3	-	119.1	36.3	-	-	-
**Py6**	102.7	-	121.7	37.2	-	-	-
**Py7**	104.1	-	121.0	37.7	-	-	-
**Im8**	-	130.1	118.9	-	23.6	23.2	49.5

Table S8. ^{13}C **PA4** chemical shifts of **PA4•ODN**4 complex.

Table S9. ^{13}C **ODN4** chemical shifts of **PA4•ODN4** complex.

ODN4	C1'	C2	C3'	C4'	C5	C6	C7	C8
**C1**	82.55	-	75.7	85.6	96.9	140.5	-	-
**G2**	81.42	-	76.4	84.7	-	-	-	135.7
**A3**	82.00	152.0	77.3	85.0	-	-	-	139.0
**T4**	83.36	-	75.5	82.8	-	136.2	-	-
**G5**	82.82	-	78.8	85.0	-	-	-	135.9
**T6**	81.42	-	73.1	79.8	-	136.5	-	-
**A7**	81.27	152.0	76.3	83.0	-	-	-	139.8
**C8**	81.60	-	72.9	80.6	96.4	139.8	-	-
**A9**	80.61	151.5	75.6	82.3	-	-	-	139.5

**T10**	81.48	-	73.0	81.7	-	135.9	-	-
**C11**	83.64	-	73.9	83.4	96.0	141.1	-	-
**G12**	82.06	-	70.8	85.3	-	-	-	136.6
**C13**	85.27	-	75.1	85.5	96.9	140.5	-	-
**G14**	81.38	-	77.2	84.7	-	-	-	137.0
**A15**	82.22	152.3	77.0	84.9	-	-	-	138.7
**T16**	82.30	-	73.2	82.2	-	136.1	-	-
**G17**	82.60	-	78.2	84.8	-	-	-	135.7
**T18**	82.00	-	74.0	81.4	-	136.3	-	-
**A19**	81.4	152.6	75.6	82.6	-	-	-	139.6
**C20**	81.55	-	72.9	80.8	96.1	139.7	-	-
**A21**	80.35	150.9	74.1	82.3	-	-	-	139.5
**T22**	81.5	-	75.0	82.0	-	135.9	-	-
**C23**	84.0	-	73.3	82.7	96.1	141.2	-	-
**G24**	82.2	-	70.9	85.3	-	-	-	135.7

Table S10. PA4•ODN4 complex minor and major groove widths; direct P-P distances and refined P-P distances, which take into account the directions of the sugar-phosphate backbones (values were calculated from a minimized average structure obtained from the clustered conformations through X3DNA; subtracting 5.8 Å from the values shown will account for the Van der Waals radii of the phosphate groups, for comparison with FreeHelix and Curves)[2].

	**Minor Groove**		**Major Groove**	
	**P-P**	**Refined**	**P-P**	**Refined**
1 CG/CG	---	---	---	---
2 GA/TC	---	---	---	---
3 AT/AT	11.6	---	16.3	---
4 TG/CA	13.6	13.6	15.7	14.9
5 GT/AC	14	14	15.1	14.6
6 TA/TA	13.5	13.4	12.7	12.6
7 AC/GT	13.7	13.6	14.7	14.7
8 CA/TG	14	---	17.6	---
9 AT/AT	---	---	---	---
10 TC/GA	---	---	---	---

Table S11. PA4•ODN4 complex axis parameters (calculated values were calculated from a minimized average structure obtained from the clustered conformations using Curves+).

**BP-Axis**	**Xdisp**	**Ydisp**	**Incline**	**Tip**	**Ax-bend**
1) G2-C23	-1.47	0.97	2.9	10.5	---
2) A3-T22	-0.57	1.61	6	4.4	2.1
3) T4-A21	0.68	1.27	10	2.9	2.2
4) G5-C20	0.35	1.24	4.2	8.9	1.8

5) T6-A19	0.54	0.14	5.6	2	1.8
6) A7-T18	0.55	-0.79	5.3	3.5	2
7) C8-G17	0.2	-1.48	6.2	-4	1.2
8) A9-T16	0.48	-0.95	6.5	-2.7	0.6
9) T10-A15	0.08	-1.73	5.6	0.3	0.5
10) C11-G14	-1.71	-1.56	5.6	-2.9	0.6
Average:	-0.09	-0.13	5.8	2.3	Total bend = 11.8 (1-10)

Table S12. PA4•ODN4 complex local base pair parameters (calculated values were calculated from a minimized average structure obtained from the clustered conformations through X3DNA).

Base pair	Shear	Stretch	Stagger	Buckle	Propeller	Opening
1 C-G	0.3	-0.15	-0.53	25.7	-6.02	-1.19
2 G-C	-0.51	-0.25	-0.24	-1.49	-9.97	-0.02
3 A-T	0.12	-0.04	0.07	4	-9.08	2.29
4 T-A	-0.29	-0.02	-0.07	15.25	-16.81	1.41
5 G-C	0	-0.09	0.07	-1.26	-0.13	-3.88
6 T-A	-0.3	0.11	0.09	6.06	-15.05	-13.63
7 A-T	-0.03	-0.06	0.16	1.89	-21.63	-9.23
8 C-G	-0.34	-0.07	0.06	-0.17	-6.88	-2.55
9 A-T	0.26	-0.07	0.24	1.45	-8.08	1.75
10 T-A	-0.15	-0.04	-0.06	12.03	6.8	-1.18
11 C-G	0.4	-0.21	-0.52	20.41	-6.14	0.04
~	~~~~~~~~~~	~~~~~~~~~~	~~~~~~~~~~	~~~~~~~~~	~~~~~~~~~~	~~~~~~~~~~
ave.	-0.05	-0.08	-0.07	7.62	-8.45	-2.38
s.d.	0.3	0.1	0.26	9.37	7.78	4.93

Table S13. PA4•ODN4 complex local base pair parameters (calculated values were calculated from a minimized average structure obtained from the clustered conformations through X3DNA).

Step	Shift	Slide	Rise	Tilt	Roll	Twist
1 CG/CG	-1.39	-1.4	4.09	-9.35	5.95	31.98
2 GA/TC	-0.48	-0.42	3.18	-3.7	-2.15	40.99
3 AT/AT	0.03	-0.48	2.98	-0.57	2.25	25.57
4 TG/CA	-2.1	0.52	3.4	-9.95	13.29	42.25
5 GT/AC	-1.03	-0.06	2.99	-1.74	-1.08	30.81
6 TA/TA	0.01	0.47	3.14	-1.27	6	36.14
7 AC/GT	0.75	0.52	3.14	2.59	-2.81	34.77
8 CA/TG	1.9	1.14	3.13	2.07	6.65	41.84
9 AT/AT	0.81	-0.67	3.28	0.03	4.83	24.8
10 TC/GA	-0.26	-0.6	3.18	1.97	0.44	35.71
~	~~~~~~~~~~	~~~~~~~~~~	~~~~~~~~~~	~~~~~~~~~	~~~~~~~~~~	~~~~~~~~~~
ave.	-0.17	-0.1	3.25	-1.99	3.34	34.49
s.d.	1.16	0.76	0.32	4.48	4.98	6.27

Table S14. Phosphate backbone angle values of **PA4•ODN4** complex (calculated values were calculated from a minimized average structure obtained from the clustered conformations through X3DNA).

Strand I

Base	alpha	beta	gamma	delta	epsilon	zeta	chi
1 C	---	---	56.9	138.5	-151.5	-130	-119.5
2 G	-77.6	-177.9	50.1	144.6	-177.4	-98.1	-127.6
3 A	-68.5	-168.1	52.9	141	-170.3	-90.8	-106.1
4 T	-73.9	169.2	53.9	139.1	-96	179	-95.9
5 G	-79.3	173.3	27.8	144.2	-179.4	-93.1	-102.1
6 T	-64.3	169.7	58.5	108.1	-175.3	-89.7	-117.3
7 A	-63.2	166.2	57.4	111.2	-176.9	-87.9	-116
8 C	-59.6	166.9	58	121.4	-166.9	-87.5	-110.5
9 A	-65.6	159.8	59.1	114.6	-171.1	-88.3	-111.9
10 T	-65.6	168.9	51	136.2	-131.2	-167.5	-103.8
11 C	-66.4	144.4	54.6	138.3	-168.7	-113	-117

Strand II

Base	alpha	beta	gamma	delta	epsilon	zeta	chi
1 G	-70.3	-178.8	51.4	122.7	---	---	-123
2 C	-66.8	176.7	59	142.6	-179.4	-89	-109.5
3 T	-60.2	165	66.5	120.3	-177	-91.5	-129
4 A	-69.1	156.8	57.1	88	176.2	-80.5	-131
5 C	-59.3	168.7	57.4	122.3	-165.8	-87	-115.2
6 A	-64.8	165.9	58.5	118.1	-179.3	-89	-114.7
7 T	-68.2	163.7	60.8	106.4	-173.6	-86.8	-117.7
8 G	-69	167.8	50.9	142.4	-174.3	-101.7	-94.8
9 T	-71.5	167.5	52.9	141.7	-129.4	-166.9	-104.8
10 A	-81.4	175.8	48.6	134.2	-148.5	-139.9	-111.2
11 G	-68.4	-170.6	58.8	138.7	-158	-77.4	-150

Table S15. NOE distance restraints of **PA4•ODN4** (values were obtained from 2D [^{1}H, ^{1}H] NOESY at 250, 200, 150, 100 ms mixing times in 99% D_2O using MARDIGRAS or at 250 ms mixing time in 90% H_2O / 10% D_2O for those involving N*H* protons; **PA4** is listed as residue 1 according to the nomenclature used in PDB 6GZ7, **ODN4** listed at residues 2-25).

ATOM-	Residue	ATOM-	Residue	low	up	center	width
H15	1	H13	1	3.78	5.64	4.71	1.86
H63	1	H53	1	4.43	5.78	5.105	1.35
H65	1	H25	1	3.62	3.75	3.685	0.13
H65	1	H63	1	3.87	4.75	4.31	0.88
H73	1	H23	1	3.3	5.92	4.61	2.62
H73	1	H63	1	3.71	4.29	4	0.58
H73	1	H75	1	3.95	5.67	4.81	1.72
M22	1	H15	1	4.55	6.23	5.39	1.68
M22	1	H25	1	2.79	3.76	3.275	0.97
M22	1	H23	1	4.43	4.73	4.58	0.3
M22	1	H73	1	4.87	5.35	5.11	0.48
M11	1	H13	1	4.12	6.12	5.12	2
M33	1	H35	1	3.74	5.13	4.435	1.39

M77	1	H25	1	3.83	5.13	4.48	1.3
M77	1	H73	1	4.53	7.03	5.78	2.5
M55	1	H53	1	4.08	5.37	4.725	1.29
M55	1	M44	1	4.29	4.33	4.31	0.04
M66	1	H63	1	4.06	6.19	5.125	2.13
H84	1	H15	1	3.81	5.81	4.81	2
H84	1	M11	1	3.84	5.1	4.47	1.26
H83	1	H13	1	3.59	7.23	5.41	3.64
H83	1	H84	1	2.73	3.67	3.2	0.94
H10	1	H15	1	3.63	3.72	3.675	0.09
H10	1	H84	1	3.42	4.36	3.89	0.94
M88	1	M11	1	4.6	6.95	5.775	2.35
M88	1	H84	1	3.11	4.19	3.65	1.08
M88	1	H83	1	4.24	6.14	5.19	1.9
M88	1	H10	1	2.84	3.4	3.12	0.56
M99	1	H15	1	3.92	5.18	4.55	1.26
M99	1	M11	1	3.56	4.72	4.14	1.16
M99	1	H84	1	2.77	3.91	3.34	1.14
M99	1	H10	1	2.84	3.89	3.365	1.05
H6	2	H4'	2	3.65	5.77	4.71	2.12
H6	2	H1'	2	3.02	4.08	3.55	1.06
H3'	2	H6	2	3.23	4.34	3.785	1.11
H2'	2	H6	2	2.37	3.19	2.78	0.82
H2''	2	H6	2	2.77	3.97	3.37	1.2
H1'	3	H4'	3	2.97	4.06	3.515	1.09
H8	3	H4'	3	3.97	4.2	4.085	0.23
H8	3	H1'	3	3.27	4.53	3.9	1.26
H2''	3	H1'	3	2.62	3.7	3.16	1.08
H8	4	H1'	3	2.99	4.08	3.535	1.09
H8	4	H8	3	3.76	5.79	4.775	2.03
H8	4	H3'	3	3.77	6.21	4.99	2.44
H8	4	H4'	4	3.82	4	3.91	0.18
H8	4	H1'	4	2.82	4.22	3.52	1.4
H3'	4	H8	4	2.92	6.68	4.8	3.76
H2''	4	H3'	4	2.61	2.89	2.75	0.28
H6	5	H1'	4	3.26	4.61	3.935	1.35
H6	5	H8	4	3.72	4.99	4.355	1.27
H6	5	H3'	4	3.73	5.31	4.52	1.58
H6	5	H2'	4	2.9	6.37	4.635	3.47
H6	5	H4'	5	3.62	6	4.81	2.38
H3'	5	H6	5	3.05	4.68	3.865	1.63
H1'	6	H83	1	2.73	3.65	3.19	0.92
H1'	6	H4'	6	3.33	4.64	3.985	1.31
H8	6	H6	5	3.68	7.86	5.77	4.18

H8	6	H4'	6	3.63	3.75	3.69	0.12
H8	6	H1'	6	3.34	4.7	4.02	1.36
H2''	6	H1'	6	2.58	3.44	3.01	0.86
H1'	7	M88	1	4.27	6.73	5.5	2.46
H1'	7	H1'	6	3.1	4.35	3.725	1.25
H1'	7	H4'	7	2.72	3.64	3.18	0.92
H6	7	H1'	6	3.16	4.37	3.765	1.21
H6	7	H8	6	3.64	5.1	4.37	1.46
H3'	7	M88	1	3.8	3.89	3.845	0.09
H3'	7	H6	7	3.17	3.56	3.365	0.39
H2''	7	H1'	7	2.49	3.17	2.83	0.68
H4'	8	H75	1	2.99	4.05	3.52	1.06
H1'	8	H73	1	2.86	4.42	3.64	1.56
H8	8	H1'	7	3.29	4.52	3.905	1.23
H8	8	H3'	7	3.32	3.99	3.655	0.67
H2	8	H1'	8	3.55	4.25	3.9	0.7
H1'	9	H63	1	2.59	3.55	3.07	0.96
H1'	9	H2	8	3.4	4.68	4.04	1.28
H1'	9	H4'	9	2.43	3.25	2.84	0.82
H6	9	H8	8	3.5	5.07	4.285	1.57
H1'	10	H53	1	2.46	3.15	2.805	0.69
H8	10	H1'	9	3.51	4.64	4.075	1.13
H8	10	H6	9	3.4	5.34	4.37	1.94
H8	10	H3'	9	3.53	4.21	3.87	0.68
H8	10	H1'	10	3.36	4.65	4.005	1.29
H2	10	H53	1	4.28	7.73	6.005	3.45
H3'	10	H8	10	3.27	6.5	4.885	3.23
H2’’	10	H1’	10	2.57	3.39	2.98	0.82
H2’’	10	H8	10	3.33	4.07	3.7	0.74
H1’	11	H4’	11	2.84	3.8	3.32	0.96
H2’’	11	H1’	11	2.58	3.05	2.815	0.47
H6	12	H1’	11	3.18	3.22	3.2	0.04
H6	12	H4’	12	3.45	4.85	4.15	1.4
H5	12	H6	11	3.52	4.19	3.855	0.67
H2’	12	H6	12	2.42	3.3	2.86	0.88
H2’’	12	H3’	12	2.51	4.05	3.28	1.54
H6	14	H4’	14	3.59	3.68	3.635	0.09
H6	14	H1’	14	3	4.12	3.56	1.12
H3’	14	H6	14	3.3	4.65	3.975	1.35
H2’	14	H6	14	2.43	3.06	2.745	0.63
H2’’	14	H6	14	3.11	4.02	3.565	0.91
H1’	15	H4’	15	3.05	4.13	3.59	1.08
H8	15	H2’	14	3.45	3.69	3.57	0.24
H8	15	H4’	15	4.02	6.47	5.245	2.45

H2’’	15	H1’	15	2.49	3.29	2.89	0.8
H8	16	H4’	16	3.88	7.57	5.725	3.69
H8	16	H1’	16	2.81	4.5	3.655	1.69
H3’	16	H8	16	3.2	4.45	3.825	1.25
H2’’	16	H8	16	2.82	3.61	3.215	0.79
H1’	17	H4’	17	2.76	3.74	3.25	0.98
H6	17	H1’	17	3.39	4.54	3.965	1.15
H3’	17	H6	17	3.21	4.32	3.765	1.11
H2’	17	H6	17	2.46	3.25	2.855	0.79
H2’’	17	H1’	17	2.44	3.38	2.91	0.94
H2’’	17	H6	17	2.86	3.91	3.385	1.05
H2’’	17	H3’	17	2.83	3.84	3.335	1.01
H8	18	H3’	17	3.59	5.32	4.455	1.73
H8	18	H4’	18	3.04	5.49	4.265	2.45
H1’	19	H4’	19	2.89	3.88	3.385	0.99
H6	19	H1’	18	3.45	3.84	3.645	0.39
H6	19	H8	18	3.6	5.29	4.445	1.69
H6	19	H1’	19	3.2	4.35	3.775	1.15
H3’	19	H6	19	2.72	3.57	3.145	0.85
H2’’	19	H1’	19	2.52	3.37	2.945	0.85
H4’	20	H35	1	3.09	4.08	3.585	0.99
H8	20	H3’	19	3.57	5.24	4.405	1.67
H2	20	H1’	20	3.6	5.27	4.435	1.67
H2’’	20	H2’	20	1.84	2.58	2.21	0.74
H1’	21	H23	1	2.59	3.5	3.045	0.91
H1’	21	H2	20	3.39	4.77	4.08	1.38
H6	21	H8	20	3.46	5.11	4.285	1.65
H4’	22	H15	1	3.09	4.12	3.605	1.03
H1’	22	H13	1	2.66	2.96	2.81	0.3
H1’	22	H4’	22	3	3.96	3.48	0.96
H8	22	H1’	21	3.66	3.77	3.715	0.11
H8	22	H6	21	3.68	4.29	3.985	0.61
H8	22	H3’	21	3.41	4.06	3.735	0.65
H8	22	H1’	22	2.83	4.53	3.68	1.7
H2	22	H2	4	3.76	3.92	3.84	0.16
H3’	22	H8	22	3.24	4.58	3.91	1.34
H2’’	22	H13	1	3.57	3.71	3.64	0.14
H2’’	22	H1’	22	2.61	3.44	3.025	0.83
H2’’	22	H8	22	2.8	3.96	3.38	1.16
H2’’	22	H3’	22	2.82	3.04	2.93	0.22
H6	23	H1’	22	3.54	4.3	3.92	0.76
H6	23	H8	22	3.7	5.46	4.58	1.76
H6	23	H2’’	22	3.07	3.2	3.135	0.13
H6	23	H1’	23	3.28	4.51	3.895	1.23

H2’’	23	H2’	23	2.28	3.17	2.725	0.89
H1’	24	H4’	24	3.09	4.55	3.82	1.46
H6	24	H1’	24	3.23	4.56	3.895	1.33
H5	24	H6	23	3.6	5.13	4.365	1.53
H2’’	24	H1’	24	2.48	3.33	2.905	0.85
H2’’	24	H6	24	3	3.34	3.17	0.34
H8	25	H1’	24	3.45	4.64	4.045	1.19
H55	1	H35	1	5.02	5.23	5.125	0.21
H33	1	H23	1	4.03	4.3	4.165	0.27
M22	1	H75	1	4.28	4.3	4.29	0.02
M11	1	H15	1	3.19	3.9	3.545	0.71
M44	1	H35	1	6.04	6.29	6.165	0.25
M44	1	H44	1	3.2	3.93	3.565	0.73
M44	1	H55	1	4.41	5.39	4.9	0.98
M77	1	H65	1	4.74	5.61	5.175	0.87
M77	1	H75	1	3.11	3.82	3.465	0.71
M55	1	H44	1	4.1	4.96	4.53	0.86
M55	1	H55	1	3.18	3.89	3.535	0.71
M66	1	H55	1	6.07	6.29	6.18	0.22
M66	1	H65	1	3.67	3.67	3.67	0
M66	1	H33	1	5.67	5.89	5.78	0.22
H83	1	M11	1	4.4	6.1	5.25	1.7
H10	1	H83	1	3.68	4.33	4.005	0.65
H2’’	3	H4’	3	4.58	4.71	4.645	0.13
H2’’	3	H3’	3	2.29	2.94	2.615	0.65
H2’	5	H6	5	1.92	2.71	2.315	0.79
H2’’	5	H6	5	1.86	2.47	2.165	0.61
H6	7	H1’	7	3.51	4.29	3.9	0.78
H2’’	7	H3’	7	1.48	2.84	2.16	1.36
H2	8	H63	1	2.77	3.48	3.125	0.71
H2	8	H33	1	2.91	3.65	3.28	0.74
H2’’	8	H1’	8	2.02	2.38	2.2	0.36
H6	9	H1’	8	4.22	4.28	4.25	0.06
H1’	10	H4’	10	1.5	1.5	1.5	0
H8	10	H53	1	4.64	4.81	4.725	0.17
H8	10	H2’’	9	1.93	2.94	2.435	1.01
H2’’	10	H2’	10	1.32	1.34	1.33	0.02
H6	11	H1’	10	4.06	4.17	4.115	0.11
H6	11	H8	10	4.34	4.51	4.425	0.17
H6	11	H1’	11	3.67	4.36	4.015	0.69
H2’’	11	H6	11	1.36	3.07	2.215	1.71
H6	12	H2’’	11	2.99	3.59	3.29	0.6
H2’’	12	H2’	12	1.5	1.56	1.53	0.06
H8	13	H4’	12	5.07	5.3	5.185	0.23

H8	13	H4’	13	4.86	5.05	4.955	0.19
H2’’	14	H2’	14	1.39	2.3	1.845	0.91
H8	15	H1’	15	3.62	4.64	4.13	1.02
H2’’	15	H3’	15	2.12	2.47	2.295	0.35
H2’	16	H8	16	2.52	3.22	2.87	0.7
H2’’	16	H3’	16	2.02	2.02	2.02	0
H2’’	16	H2’	16	2.23	2.87	2.55	0.64
H8	18	H1’	17	3.54	4.61	4.075	1.07
H2’’	18	H4’	18	3.31	4.26	3.785	0.95
H6	19	H3’	18	4.09	7.54	5.815	3.45
H1’	20	H33	1	2.97	3.76	3.365	0.79
H1’	20	H4’	20	2.02	3.12	2.57	1.1
H2’’	21	H1’	21	1.78	2.31	2.045	0.53
H8	22	H2’’	21	2.12	3	2.56	0.88
H8	22	H4’	22	3.29	3.92	3.605	0.63
H1’	23	H4’	23	2.51	3.11	2.81	0.6
H8	8	H6	7	3.94	4.09	4.015	0.15
H8	8	H1’	8	3.6	3.68	3.64	0.08
H2	8	H23	1	3.89	4.04	3.965	0.15
H5	9	H8	8	3.54	3.73	3.635	0.19
H3’	9	H6	9	3.29	3.33	3.31	0.04
H2’’	11	H2’	11	2.23	2.37	2.3	0.14
H1’	12	H4’	12	3.02	3.04	3.03	0.02
H2’’	12	H6	12	3.02	3.04	3.03	0.02
H8	13	H1’	13	3.49	3.82	3.655	0.33
H2’’	14	H3’	14	3.02	3.41	3.215	0.39
H8	16	H2’	15	1.94	1.94	1.94	0
H3’	18	H8	18	3.88	4.07	3.975	0.19
H8	20	H1’	19	3.82	3.92	3.87	0.1
H8	20	H6	19	3.76	3.88	3.82	0.12
H8	20	H1’	20	3.3	3.99	3.645	0.69
H2	20	H63	1	3.65	4.12	3.885	0.47
H5	21	H8	20	3.4	3.45	3.425	0.05
H3’	21	H6	21	3.41	3.46	3.435	0.05
H8	25	H2’’	24	3.71	3.83	3.77	0.12
H8	25	H1’	25	3.69	3.88	3.785	0.19
H33	1	H35	1	4.2	4.54	4.37	0.34
H6	9	H1’	9	3.58	3.69	3.635	0.11
H13	1	HN03	1	2.25	2.26	2.255	0.01
H25	1	HN2	1	3.43	3.58	3.505	0.15
HN4	1	HN3	1	3.19	3.28	3.235	0.09
H23	1	HN2	1	2.51	2.53	2.52	0.02
HN9	1	HN4	1	3.08	3.16	3.12	0.08
H35	1	HN3	1	3.43	3.57	3.5	0.14

HN6	1	HN2	1	3.38	3.51	3.445	0.13
HN6	1	H23	1	3.48	3.7	3.59	0.22
HN7	1	H13	1	3.39	3.54	3.465	0.15
HN7	1	HN1	1	3.39	3.56	3.475	0.17
HN7	1	H23	1	3.41	3.59	3.5	0.18
H44	1	HN4	1	3.43	3.57	3.5	0.14
H53	1	HN3	1	3.35	3.49	3.42	0.14
H53	1	HN4	1	2.98	3.03	3.005	0.05
H53	1	HN9	1	2.2	2.21	2.205	0.01
H53	1	HN5	1	2.52	2.54	2.53	0.02
H55	1	HN5	1	3.39	3.49	3.44	0.1
H63	1	HN5	1	2.17	2.18	2.175	0.01
H63	1	HN6	1	2.37	2.38	2.375	0.01
H65	1	HN6	1	3.34	3.44	3.39	0.1
H75	1	HN7	1	3.34	3.46	3.4	0.12
H33	1	HN2	1	2.19	2.2	2.195	0.01
H33	1	HN3	1	2.4	2.41	2.405	0.01
H73	1	HN1	1	3.41	3.59	3.5	0.18
H73	1	HN2	1	3.25	3.36	3.305	0.11
H73	1	HN7	1	2.4	2.42	2.41	0.02
H83	1	HN7	1	3.37	3.5	3.435	0.13
M88	1	HN7	1	4.31	4.55	4.43	0.24
H1	6	HN03	1	3.54	3.77	3.655	0.23
H1	6	H13	1	2.87	2.91	2.89	0.04
H1	6	HN1	1	3.49	3.67	3.58	0.18
H1	6	H23	1	3.43	3.63	3.53	0.2
H1	6	H83	1	3.5	3.65	3.575	0.15
H21	6	H1	6	2.26	2.27	2.265	0.01
H22	6	H1	6	3.27	3.32	3.295	0.05
H4’	8	HN7	1	3.41	3.56	3.485	0.15
H1’	8	HN6	1	2.35	2.36	2.355	0.01
H1’	8	HN7	1	3.32	3.45	3.385	0.13
H2	8	HN3	1	3.02	3.08	3.05	0.06
H2	8	HN5	1	2.93	2.99	2.96	0.06
H1’	9	HN5	1	2.34	2.36	2.35	0.02
H1’	9	HN6	1	3.05	3.12	3.085	0.07
H1’	10	HN5	1	3.07	3.14	3.105	0.07
H2	10	HN4	1	3.4	3.53	3.465	0.13
H2	10	HN9	1	2.49	2.5	2.495	0.01
H1	15	H41	12	3.15	3.25	3.2	0.1
H1	15	H42	12	3.22	3.34	3.28	0.12
H2	16	H3	11	2.5	2.51	2.505	0.01
H3	17	HN9	1	3.64	3.88	3.76	0.24
H3	17	H5	9	3.76	4.11	3.935	0.35

H3	17	H2	10	2.34	2.35	2.345	0.01
H1’	18	HN4	1	2.51	2.52	2.515	0.01
H1	18	HN4	1	3.35	3.49	3.42	0.14
H1	18	H5	9	3.62	3.86	3.74	0.24
H1	18	H41	9	2.44	2.45	2.445	0.01
H1	18	H42	9	2.47	2.49	2.48	0.02
H1	18	H3	17	3.42	3.57	3.495	0.15
H21	18	HN4	1	2.71	2.74	2.725	0.03
H21	18	HN5	1	2.82	2.86	2.84	0.04
H21	18	H53	1	2.49	2.51	2.5	0.02
H21	18	H63	1	3.04	3.11	3.075	0.07
H21	18	H1	18	2.38	2.39	2.385	0.01
H22	18	HN4	1	2.55	2.57	2.56	0.02
H22	18	HN9	1	2.94	3	2.97	0.06
H22	18	HN5	1	2.87	2.91	2.89	0.04
H22	18	H2	8	3.14	3.22	3.18	0.08
H22	18	H1	18	2.49	2.51	2.5	0.02
H22	18	H21	18	2.22	2.23	2.225	0.01
H1’	19	HN3	1	2.22	2.23	2.225	0.01
H1’	19	HN4	1	3.53	3.66	3.595	0.13
H1’	21	HN1	1	2.34	2.35	2.345	0.01
H1’	21	HN2	1	3.16	3.24	3.2	0.08
H41	21	H1	6	2.37	2.39	2.38	0.02
H42	21	H1	6	2.81	2.88	2.845	0.07
H4’	22	HN1	1	3.11	3.2	3.155	0.09
H1’	22	HN03	1	2.49	2.51	2.5	0.02
H1’	22	HN1	1	3.26	3.39	3.325	0.13
H2	22	HN03	1	2.56	2.57	2.565	0.01
H1’	23	HN03	1	3.14	3.23	3.185	0.09
H3	23	H2	4	2.57	2.59	2.58	0.02
H41	24	H1	3	3.37	3.5	3.435	0.13
H42	24	H1	3	3.2	3.29	3.245	0.09
H3	7	H22	6	3.38	3.45	3.415	0.07

Total: 310 distance restraints

3.0 Supplementary methods

3.1 General procedures

All reagents and solvents from commercial suppliers were used without further purification, unless otherwise stated. Solvents were of HPLC or peptide grade and used without further purification, unless otherwise stated. NMR spectroscopic analysis of monomers were performed on a Bruker AV 500 MHz spectrometer. All chemical shifts (δ) were referenced to the singlet methyl signal of 3-trimethylsilyl-2,2,3,3-d4 propanoic acid sodium salt (TSP-d4) and are reported in parts-per-million (ppm). Coupling constants are quoted in Hertz (Hz). Abbreviations for splitting patterns are: s - (singlet), d -(doublet), t - (triplet), q - (quartet), sep - (septet), and m - (multiplet). NMR data were processed using Topspin 3.5pl7. Purification of polyamides was carried out on a Dionex UltiMate 3000 HPLC instrument utilizing a Kinetex 5 µm C-18 reverse-phase column (dimensions 150 x 21.2 mm). Analytical HPLC traces were recorded on a Shimadzu Prominence HPLC using Aeris 3.6 µm Widepore XB-C18 (size 250 x 4.6 mm). ATR-IR data was collected on an Agilent spectrometer and the data processed using Spectrum One software (only major absorbances are reported). Melting points of solids were obtained using a Griffin melting point apparatus. Polyamide **PA4** and monomer **^{i}Pr-Im-COCCl$_3$** HRMS data were obtained from the U.K. National Mass Spectrometry Facility at Swansea University. *Chemicalize* was used for prediction of **PA1-4** logD values, Jul, 2018, (https://chemicalize.com/ developed by ChemAxon (http://www.chemaxon.com)). Pyrrole and imidazole monomer blocks (Py, Im) were synthesized according to published literature procedures.[3,4] Structural images for the 3D complexes and free DNA were obtained from the PDB with ID codes 5OCZ, 5OE1, 5ODF, and 5ODM for the ODN4, along with PA1-3•ODN4 complexes and Chimera.

Synthesis of *N*-isopropyl-2-trichloroacetyl imidazole (^{i}**Pr-Im-COCCl$_3$**)

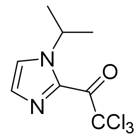

To a solution of trichloroacetyl chloride (5.5 g, 30.2 mmol) in 8 mL methylene chloride was charged a solution of *N*-isopropylimidazole (3.3 g, 30.0 mmol) in 15 mL methylene chloride over a period of 2.5 h via syringe pump at ambient (19-25 °C). The resulting solution was stirred with a magnetic stir bar at ambient for 17 h, at which point the reaction mixture was cooled to 5 °C and triethylamine was added, forming a precipitate (TEA•HCl). The slurry was allowed to warm to ambient and stirred for a further 2 h prior to filtration through a 55 mm diameter Buchner funnel lined with filter paper. The filtrate was distilled to dryness under reduced pressure and the oily concentrate flash-purified through normal phase silica gel chromatography (5:1 DCM/petroleum ether, R_f = 0.57) to give ^{i}**Pr-Im-COCCl$_3$** as an off-white solid (3.60 g, 47% yield). The monomer was stored in a freezer (-15 to -20 °C) until required for coupling.

2-trichloroacetyl-N-isopropyl imidazole (^{i}**Pr-Im-COCCl$_3$**) (yield 47%)

Melting point = 79-80 °C

^{1}H NMR ($CDCl_3$ 500 MHz) δ: 1.45 (d, 6H, *J* = 6.7 Hz), 5.34 (sep, 1H, *J* = 6.7 Hz), 7.31 (s, 2H)

^{13}C NMR ($CDCl_3$ 126 MHz) δ: 172.3, 135.4, 130.9, 123.0, 95.3, 50.4, 23.6

IR (neat) u_{max}: 1690 cm^{-1}, 1385 cm^{-1}, 1364 cm^{-1}

ESI (+ve mode) HRMS calculated for $[M+H]^+$ 254.9857; found 254.9853.

^{1}H NMR spectrum of ^{i}**Pr-Im-COCCl$_3$**

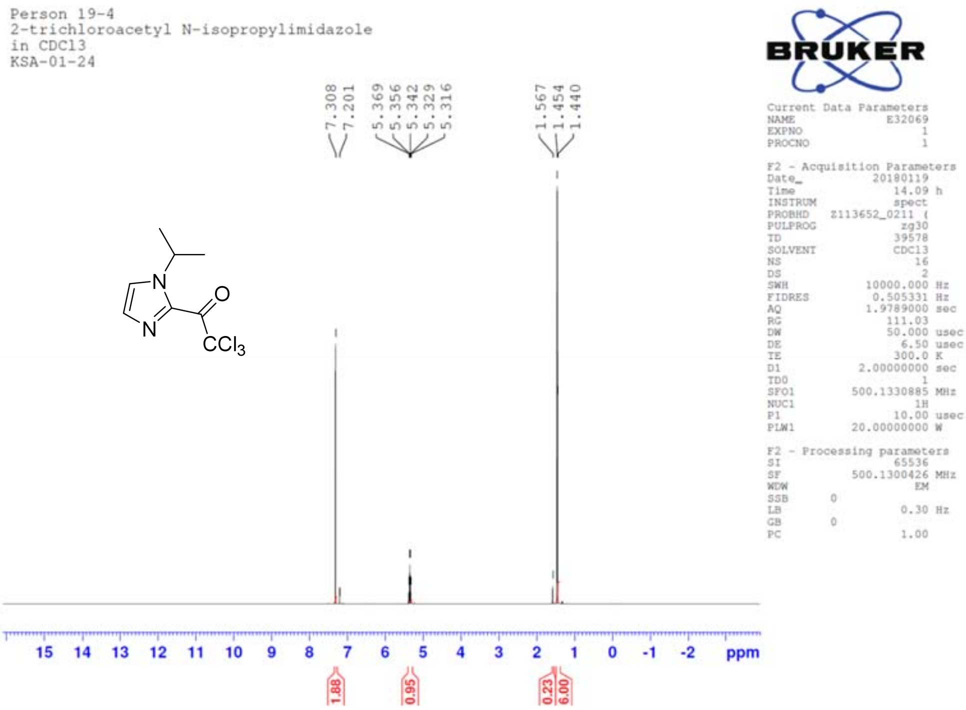

^{13}C NMR of ^{i}**Pr-Im-COCCl$_3$**

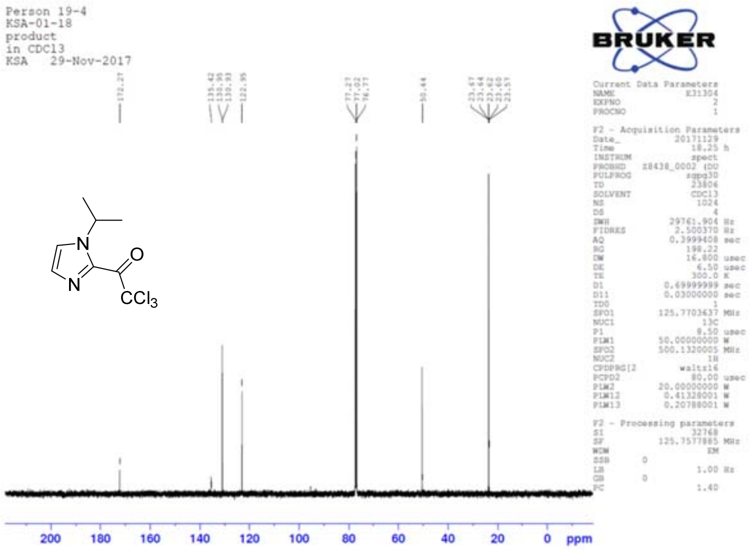

ATR-IR spectrum of ^{i}**Pr-Im-COCCl$_3$**

Agilent Technologies

Sample ID:	KSA-01-24	Method Name:	ATR 32 4cm
Sample Scans:	32	User:	STUDENT
Background Scans:	32	Date/Time:	03/04/2018 16:41:55
Resolution:	4 cm-1	Range:	4,000.00 - 650.00
System Status:	Good	Apodization:	Happ-Genzel
File Location:	C:\Program Files (x86)\Agilent\MicroLab PC\Results\ATR 32 4cm\KSA-01-24_2018-04-03T16-43-30.a2r		

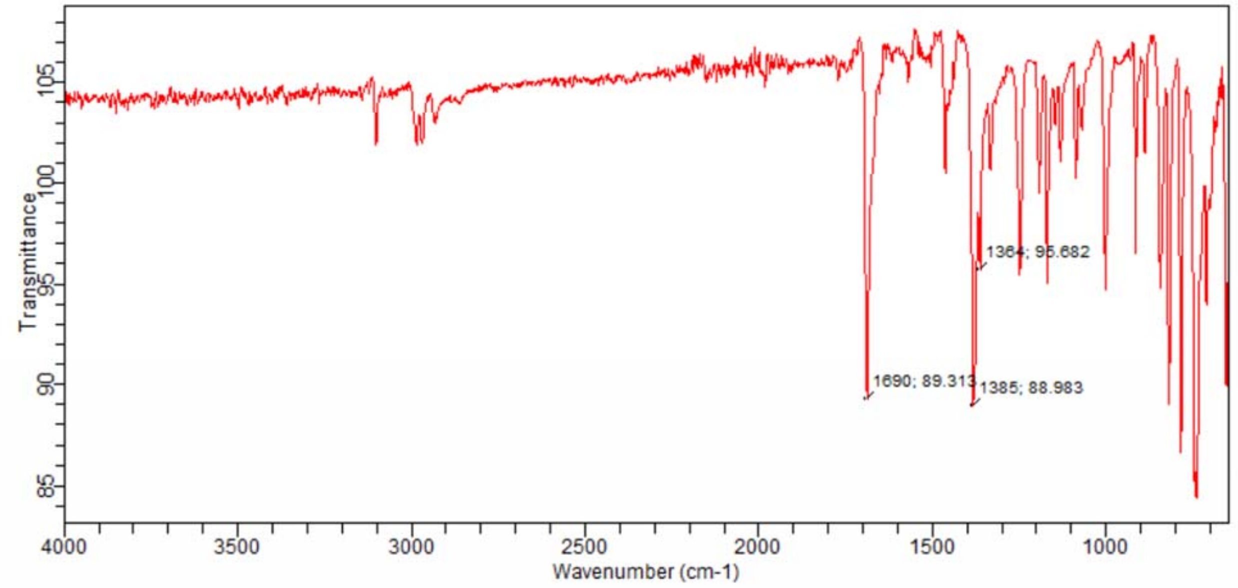

ESI-HRMS spectrum of **^{i}Pr-Im-COCCl$_3$**

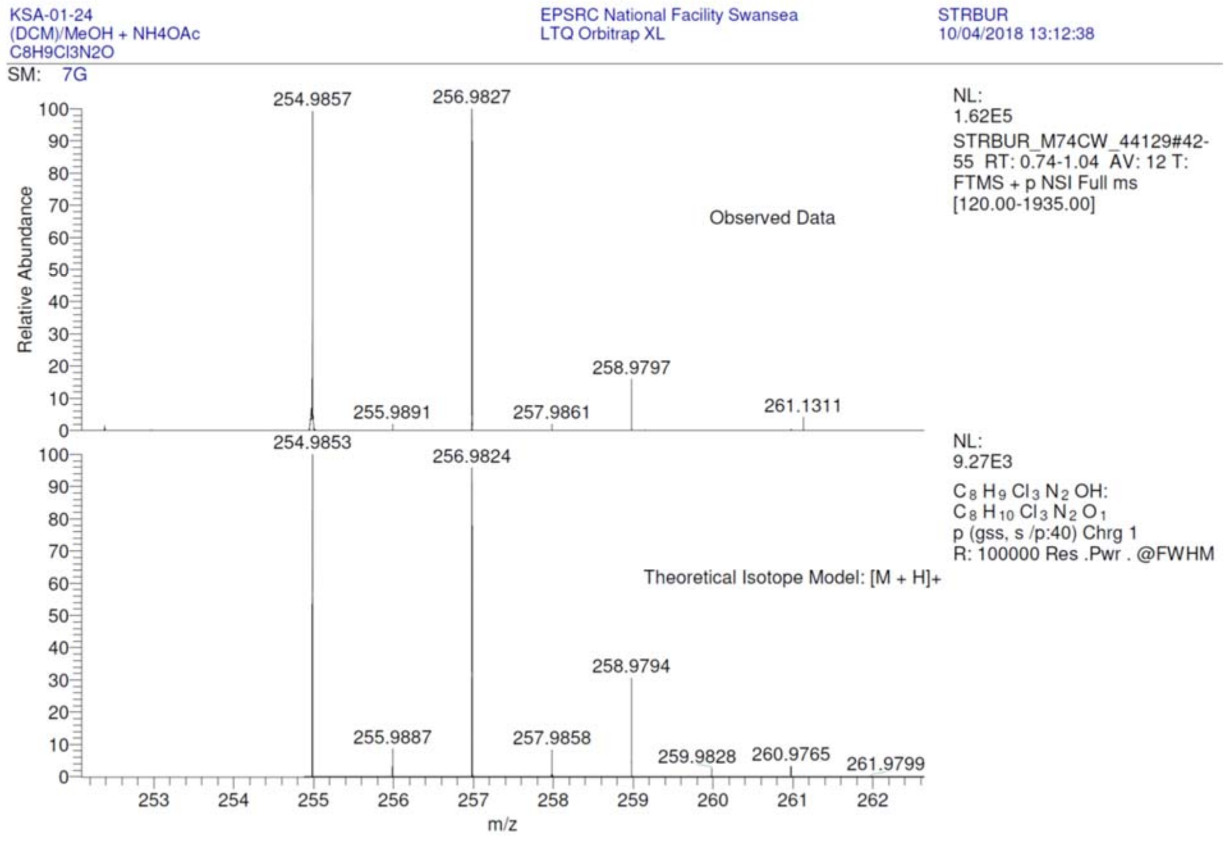

3.2 Synthesis of **PA4**.

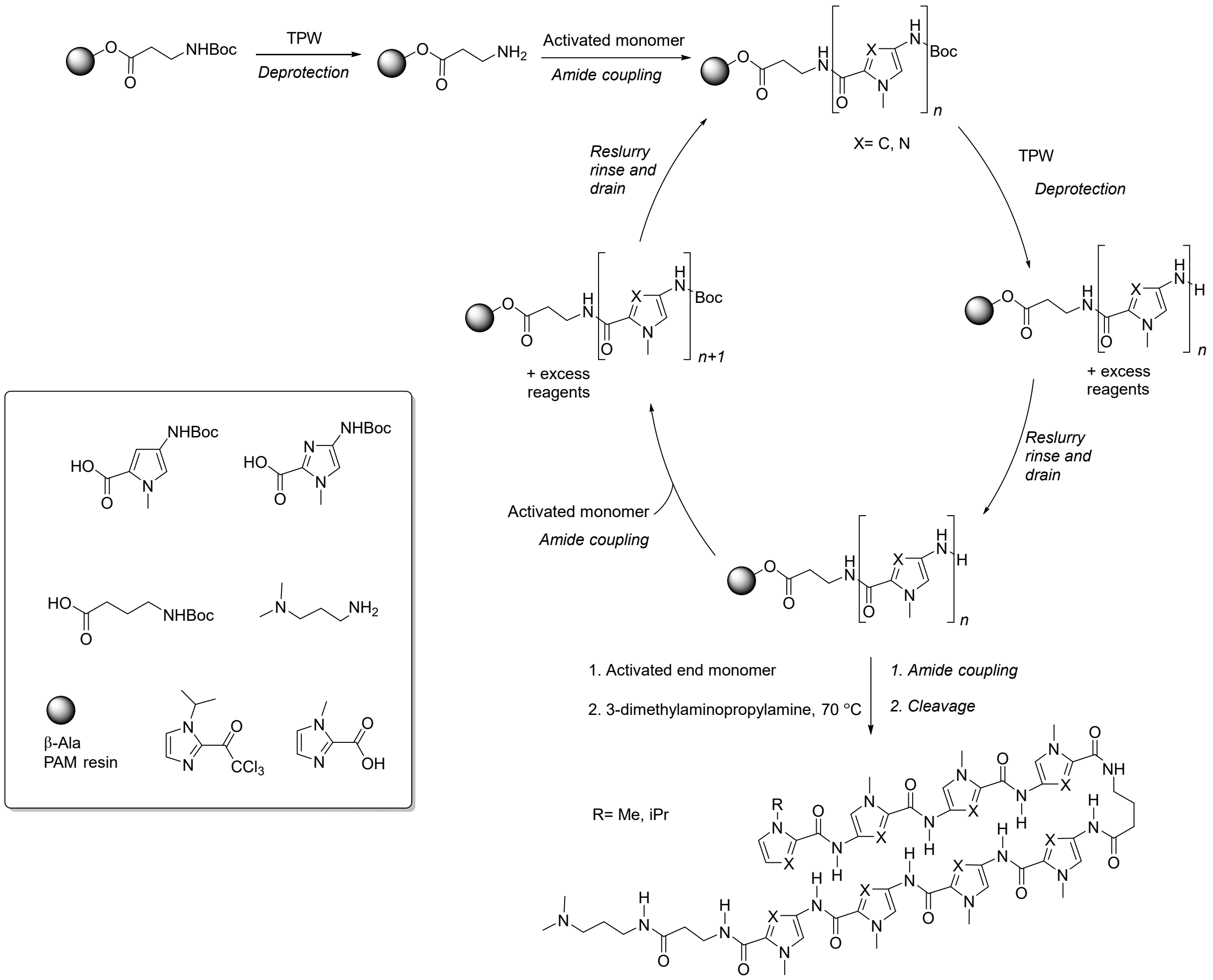

Scheme S1. Solid phase synthesis of **PA4**.

Procedure for synthesis of **PA4**

PA4 was prepared starting from the Boc-β-Ala-PAM resin (~0.5 mmol/g loading, supplier specified). After 1 h swelling of the resin in methylene chloride, deprotection of the Boc group was achieved with a TPW mixture (92.5% TFA, 5% phenol, 2.5% water) – 1 x 1 min + 2 x 3 min for Py and turn units, 1 x 1min + 2 x 10 min for Im unit.

Py/Py and GABA/Py coupling

Pre-activation of monomer unit: 4.0 mol equiv of Py with 3.6 equiv HATU in 1.5 mL anhydrous DMF. 9 equiv DIPEA added and the mixture agitated for 20 minutes at ambient (19-25 °C).

After deprotection, the resin was washed with DMF (4 x 1 mL x 1 min), rinsed with 1.5 mL anhydrous DMF. The pre-activated mixture of Py (4 mol equiv) in 1.5 mL anhydrous DMF with 3.6 equiv HATU and 9 equiv DIPEA was charged to the resin. The resulting slurry was allowed to agitate for not less than 2 h at ambient (19-25 °C).

Py/Im, Im/GABA coupling

Pre-activation of monomer unit: 4.0 mol equiv of Im or GABA with 3.6 equiv DCC, 3.8 equiv HOAt in 1.5 mL anhydrous DMF for 2 h, filtration of DCU precipitate, and addition of 9 equiv DIPEA prior to coupling.

After deprotection, the resin was washed with DMF (4 x 1 mL x 1 min), followed by rinsing with 1.5 mL anhydrous DMF. The pre-activated mixture of Im or GABA (4 mol equiv) was charged to the resin and allowed to agitate for a minimum 12 h at ambient (19-25 °C).

N-isopropylimidazole *N*-terminal coupling

After deprotection, the resin was washed with DMF (4 x 1 mL x 1 min), rinsed with 1.5 mL anhydrous DMF, at which point a solution of the ^{i}PrIm-$COCCl_3$ monomer in 1.5 mL anhydrous DMF with 9 equiv DIPEA was charged to the resin and the resulting mixture agitated at ambient (19-25 °C) for 12 h.

Protocol for cleavage of polyamide from resin

Polyamides covalently attached to resin were agitated in 1.5 mL of 3-dimethylaminopropylamine at 70 °C for 16 h. After cooling to ambient (19-25 °C), the resin was filtered off and washed with 2 x 0.5 mL methanol. The resulting solution was purified through semi-prep reverse-phase HPLC.

Polyamide **PA4**

Isolated yield (from starting Boc-β-Ala PAM resin): 11%

^{1}H NMR (d6-DMSO 500 MHz) δ: 1.42 (d, 6H, J=6.65 Hz), 1.71-1.83 (m, 4H), 2.34-2.38 (m, 4H), 2.62-2.65 (m, 2H), 2.74 (s, 3H), 2.75 (s, 3H), 2.99-3.03 (m, 1H), 3.10-3.14 (m, 1H), 3.19-3.23 (m, 1H), 3.80 (s, 6H), 3.85-3.86 (m, 12H), 3.96 (s, 3H), 5.59-5.65 (sep, 1H, J = 6.65 Hz), 6.88 (s, 1H), 6.90 (s, 1H), 7.07-7.08 (m, 3H), 7.16-7.17 (m, 4H), 7.22 (s, 1H), 7.23 (s, 1H), 7.27 (s, 1H), 7.30 (s, 1H), 7.46 (s, 1H), 7.62 (s, 1H), 8.01-8.03 (m, 2H), 9.18 (s, 1H), 9.88 (s, 1H), 9.89 (s, 1H), 9.93 (s, 1H), 9.94 (s, 1H), 9.96 (s, 1H) 10.24 (s, 1H), 10.43 (s, 1H).

HPLC purity (310 nm): 95.7% [retention time: 21.66 min]

ESI-HRMS: 1250.6059 [1250.6079 calculated for M+H $C_{58}H_{72}N_{21}O_{10}^{+}$]

^{1}H-NMR (d_6-DMSO) spectrum of **PA4**

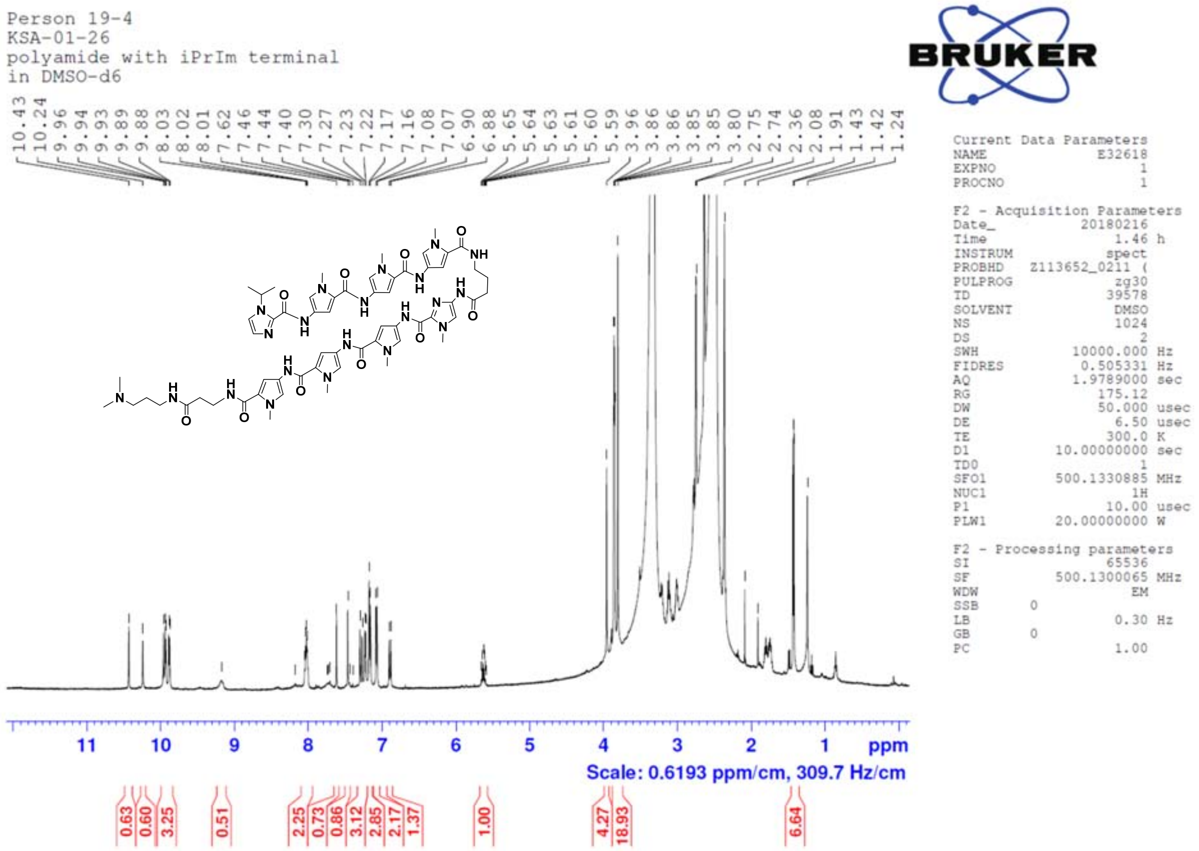

HPLC purity (λ = 310 nm) of **PA4.**

SHIMADZU LabSolutions **Analysis Report**

<Sample Information>

Sample Name	: KSA_01_20_A_II		
Sample ID	: KSA_01_20_A_II		
Data Filename	: polyamide with isopropyl_II_15122017_005.lcd		
Method Filename	: PA Method trial.lcm		
Batch Filename	: polyamide with isopropyl_II.lcb		
Vial #	: 1-2	Sample Type	: Unknown
Injection Volume	: 50 uL		
Date Acquired	: 15/12/2017 17:19:00	Acquired by	: Shimadzu
Date Processed	: 15/12/2017 18:02:02	Processed by	: Shimadzu

<Chromatogram>

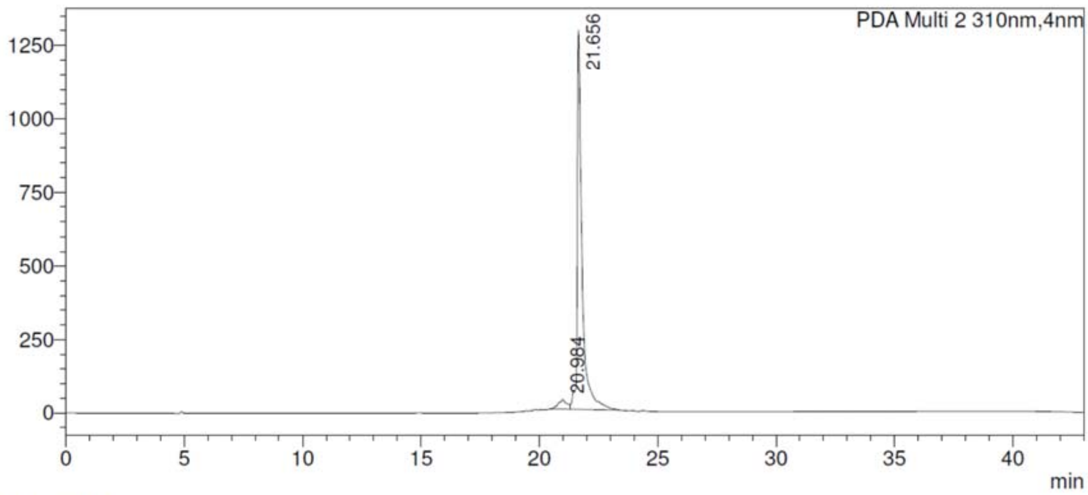

<Peak Table>

PDA Ch2 310nm

Peak#	Ret. Time	Area	Height	Conc.	Unit	Mark	Name
1	20.984	805317	33204	4.286		M	
2	21.656	17983828	1289934	95.714		V M	
Total		18789144	1323138				

ATR-IR spectrum of **PA4**.

Agilent Technologies

Sample ID:	KSA-01-26	Method Name:	ATR 32 4cm
Sample Scans:	32	User:	STUDENT
Background Scans:	32	Date/Time:	03/04/2018 16:55:38
Resolution:	4 cm-1	Range:	4,000.00 - 650.00
System Status:	Good	Apodization:	Happp-Genzel

File Location: C:\Program Files (x86)\Agilent\MicroLab PC\Results\ATR 32 4cm\KSA-01-26_2018-04-03T16-57-07.a2r

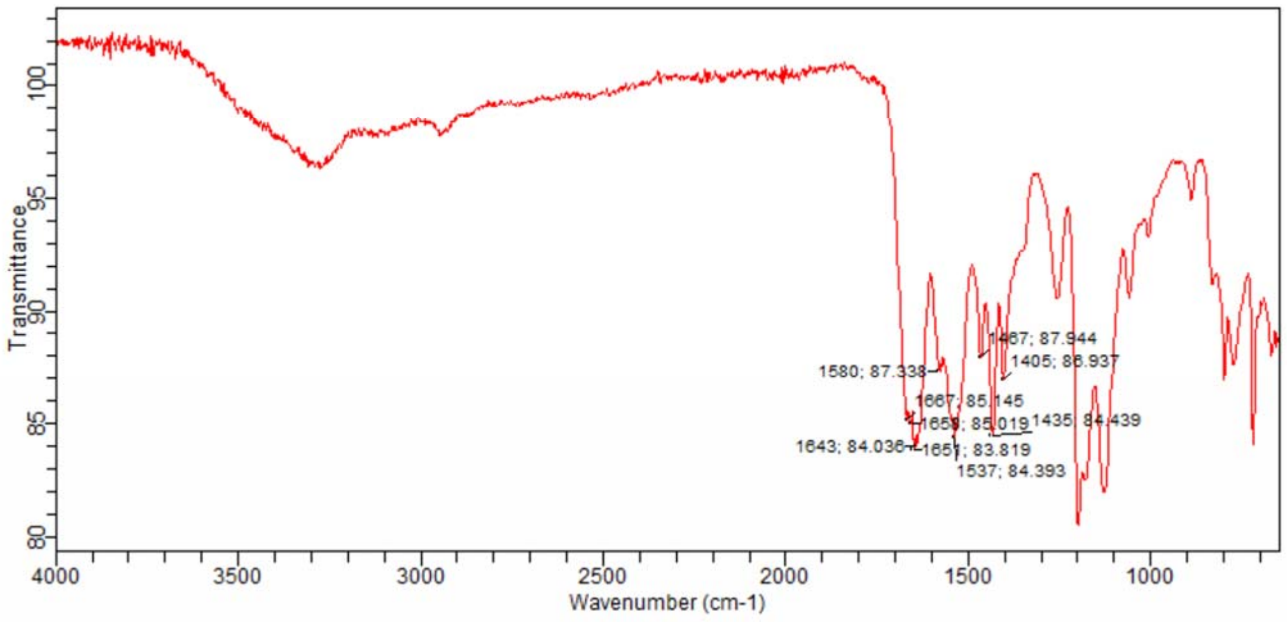

HRMS of **PA4**.

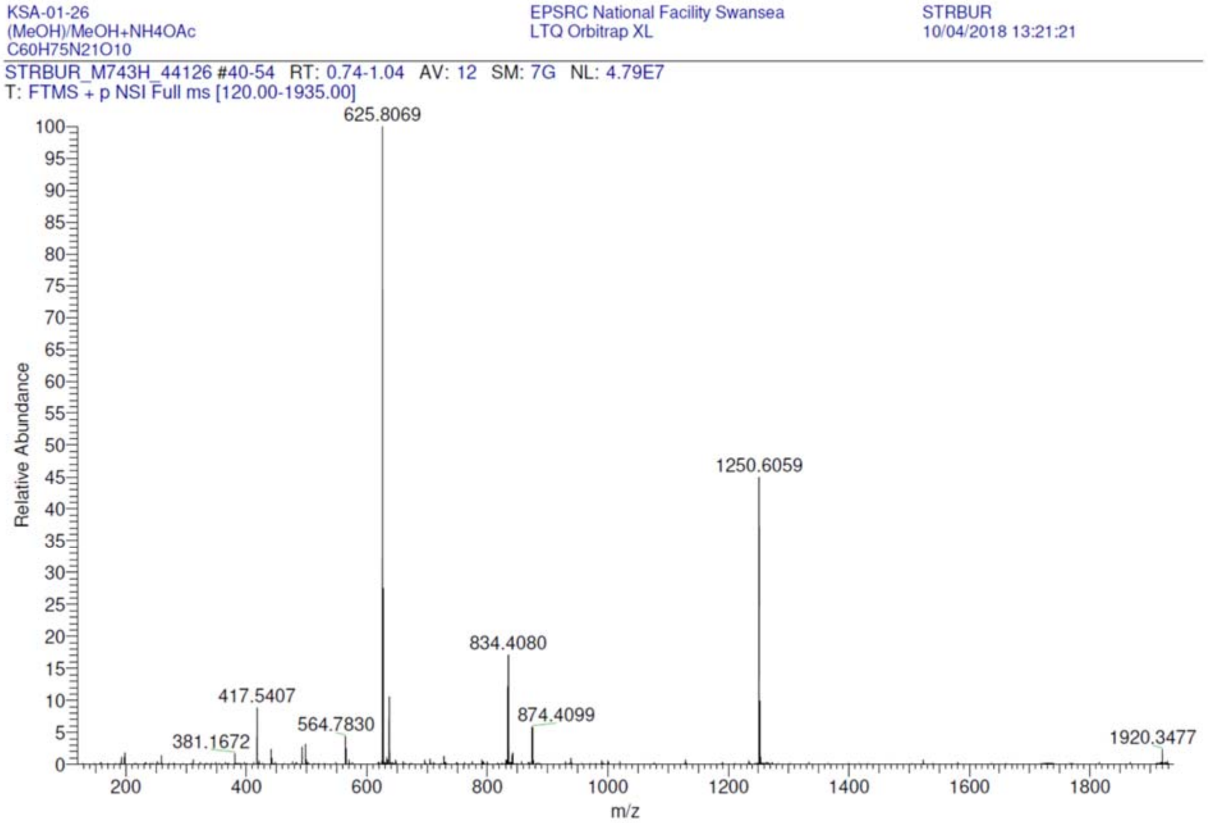

KSA-01-26
(MeOH)/MeOH+NH4OAc
C60H75N21O10
EPSRC National Facility Swansea
LTQ Orbitrap XL
STRBUR
10/04/2018 13:21:21
STRBUR_M743H_44126 #40-54 RT: 0.74-1.04 AV: 12 SM: 7G NL: 4.79E7
T: FTMS + p NSI Full ms [120.00-1935.00]
625.8069
1250.6059
834.4080
417.5407
874.4099
564.7830
381.1672
1920.3477
Relative Abundance
m/z

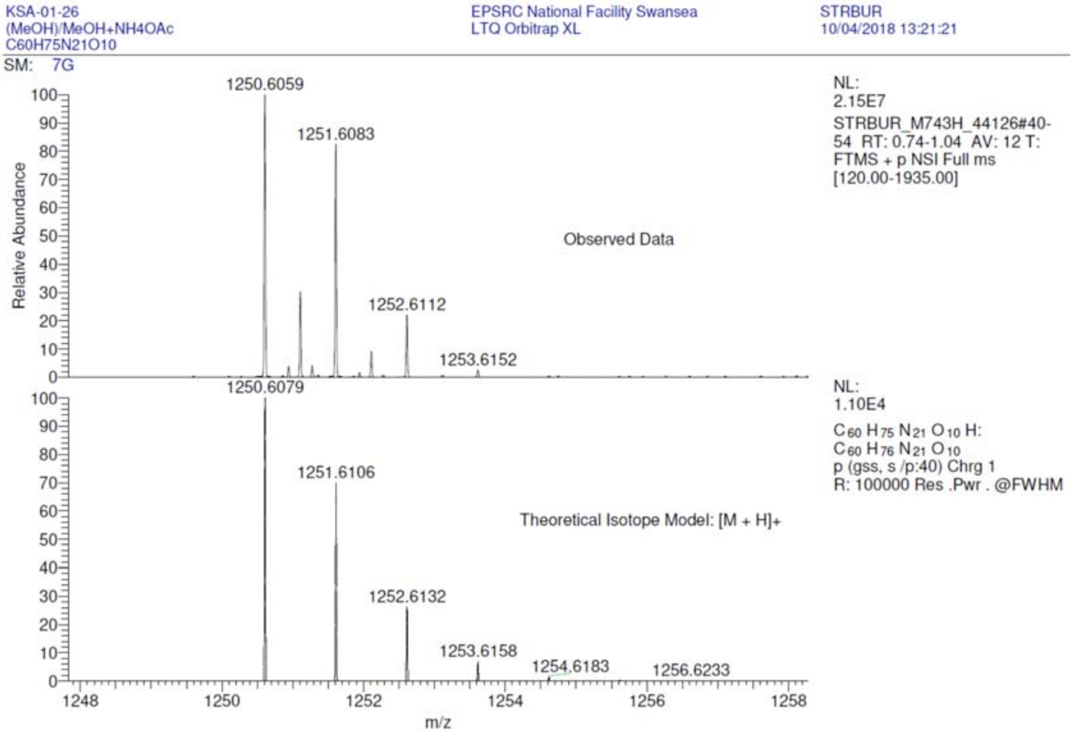

3.3 Dynamic Biosensors switchSENSE® methods

Pre-hybridization of DNA probes for kinetics experiments

DNA oligos were produced containing – at the 3'-end – a generic 48 mer switchSENSE immobilization sequence (5' - ATC AGC GTT CGA TGC TTC CGA CTA ATC AGC CAT ATC AGC TTA CGA CTA- 3'), complementary to the tethered DNA on the switchSENSE chip, and the corresponding target sequence as an overhang at the 5’-end. Short oligos complementary to the individual overhang sequences were also synthesized. All DNA oligos used for switchSENSE experiments were synthesized by biomers.net GmbH (Ulm, Germany).

To yield DNA probes with double-stranded overhang regions, the immobilization strand and the corresponding overhang complement strand were mixed at an equal concentration (500 nM) in PE140 buffer (10 mM NaPi, pH 7.4, 140 mM NaCl, 50 µM EDTA, 50 µM EGTA, 0.05 % Tween 20) and heated to 70°C. For optimal annealing, the DNA solution mix was then slowly cooled to room temperature at a rate of 1 °C/min.

For immobilization of DNA probes on switchSENSE chips, the pre-hybridized DNA probes were hybridized to the surface grafted complement of the immobilization sequence using an inbuilt routine of the DRX instrument.

Kinetics determination

Binding experiments of polyamides to DNA probes were carried out using a DRX switchSENSE platform on an MPC2-48-2-Y1-S sensor chip (Dynamic Biosensors GmbH, Martinsried, Germany). PE140 buffer (10 mM NaPi, pH 7.4, 140 mM NaCl, 50 µM EDTA, 50 µM EGTA, 0.05 % Tween 20) served as running buffer for the interaction experiments and for hybridization and on-chip immobilization of the DNA nano-levers.

The association and the dissociation kinetics of the polyamides to the DNA probes was measured under a flow rate of 1000 µl/min polyamide solution and buffer, respectively. Binding traces, corresponding to the absolute fluorescence intensity readout, were recorded using the instrument's **static measurement mode**. In this measurement mode, a constant mild negative potential is applied to the measurement electrode, which results in an upright orientation of the DNA nano-levers. Every set of association and dissociation experimental cycles in the polyamide concentration series was referenced to a buffer blank injection recorded prior to the polyamide injection. The sensor surface was regenerated after every dissociation measurement, by treatment with a basic regeneration solution (Dynamic Biosensors GmbH, Martinsried, Germany), allowing for the immobilization of fresh DNA probes for the subsequent measurements.

For data analysis, the kinetic data sets were processed using switchANALYSIS software (Dynamic Biosensors GmbH, Martinsried, Germany) and finalized plots were generated with Origin2015® software (Additive GmbH, Friedrichsdorf, Germany).

The kinetic rate constants were determined with the following equations:

Dissociation : $y(t) = y_0 + A_{off} * e^{-\frac{t}{\tau_{off}}}$ $\quad$ **Equation S1.**

Where $k_{off} = \frac{1}{\tau_{off}}$

Association : $y(t) = y_0 + A_{on} * e^{-t*(c*k_{on}+k_{off})}$ $\quad$ **Equation S2.**

y = sensor signal
y_0 = y offset
A_{off} = fit amplitude of dissociation fit
t = time; τ_{off} = dissociation time constant
k_{off} = dissociation rate constant
A_{on} = fit amplitude of association fit
c = concentration
k_{on} = association rate constant.

Equilibrium Dissociation constant : $K_D = \frac{k_{off}}{k_{on}}$ $\quad\quad$ $\boldsymbol{Equation\ S3.}$

Melting temperature determination

Melting experiments were carried out using a DRX switchSENSE platform on a MPC2-48-2-Y1-S sensor chip (Dynamic Biosensors GmbH, Martinsried, Germany). PE140 buffer (10 mM NaPi, pH 7.4, 140 mM NaCl, 50 µM EDTA, 50 µM EGTA, 0.05 % Tween 20) served as running buffer for the melting experiments and on-chip immobilization of the pre-hybridized DNA probes.

Pre-hybridized DNA probes, both in the absence and in the presence of 20 nM of polyamide, were immobilized on the switchSENSE chip and heated in PE140 buffer to 60 °C at a rate of 5 °C/min, under a buffer flow rate of 5 µl/min. The melting curves were recorded in the static measurement mode and referenced to the temperature-dependent fluorescence intensity change of the DNA-attached dye.

The melting curves were referenced to the bare DNA measurements using the switchANALYSIS software (Dynamic Biosensors GmbH, Martinsried, Germany). Final data processing was carried out using Origin2015® software (Additive GmbH, Friedrichsdorf, Germany). For better visibility of the thermal shift, the referenced data was normalized. The melting temperatures were extracted from the referenced and normalized data by global non-linear regression using a Boltzmann equation:

$$y = \frac{A_1 - A_2}{1 + e^{(T-T_M)/dx}} + A_2 \qquad \textbf{Equation S4.}$$

y = sensor signal

A_1 = sensor signal start level

A_2 = sensor signal saturation level

T = Temperature

T_M = Melting Temperature

To quantify the degree of thermal stabilization, the thermal shift (ΔT_M) was calculated from the melting temperatures in presence of polyamide and absence of polyamide.

$$\Delta T_M = T_{M-20\,nM} - T_{M-0\,nM} \qquad \textbf{Equation S5.}$$

$T_{M-20\,nM}$ = Melting Temperature at 20 nM polyamide concentration

$T_{M-0\,nM}$ = Melting Temperature at 0 nM polyamide concentration

3.4 NMR methods

NMR Sample Preparation

A sample of HPLC purified self-complementary $d(CGATGTACATCG)_2$ (**ODN4**) was purchased from Eurogentec and dissolved in 600 µL of H_2O buffered with 100 mM phosphate at pH = 7.4 containing 0.1 mM deuterated trimethylsilylpropionic acid sodium salt-*d*4 and lyophilized. The sample was re-dissolved in 600 µL of 90% H_2O with 10% D_2O. A 5 mm high-precision NMR tube was used for ODN4 data acquisition. A concentrated solution of **PA4** (15 mM in Milli-Q water) was added in small aliquots. After each addition of polyamide to the buffered solution containing **ODN4**, 1D ^1H NMR spectra were acquired until the end point of the titration was reached as determined by inspection of the imino proton chemical

shift region. After acquisition of the NOESY spectrum in 10% D_2O / 90% H_2O, the sample was lyophilized and re-dissolved in 99% D_2O to complete the acquisition of the full data set (HSQC, TOCSY, and NOESY at 4 mixing times).

NMR Data Acquisition

NMR data for polyamide-DNA complex was collected on a Bruker AVANCE-II$^+$ NMR spectrometer operating at a magnetic field of 14.1 T (600.13 MHz for ^{1}H resonance). Software for running the NMR experiment, including data collection, was TopSpin (version 3.5 patch level 5, Bruker, Reinstetten, Germany) running on a Hewlett Packard Z420 workstation under Windows Professional version 7 (Microsoft Inc.). A triple-resonance probe head [TBI-z] equipped with actively shielded z-gradient coil for delivery of pulsed field gradients was manually tuned for a [^{1}H, ^{13}C, and ^{31}P] frequency configuration on each sample studied to allow uninterrupted data acquisition to progress using all channels in their turn. The probe temperature was maintained at 298 K in all instances. One-dimensional (1D) ^{1}H NMR data were acquired using either pre-saturation (pulse program zggppr) or double pulsed field gradient spin-echo (pulse program zgesgp) to eliminate the residual solvent resonance. Data were typically acquired for 90% H_2O samples with between 64 and 256 transients into 32K data points (acquisition time: 1.09 s) over a frequency width equivalent to 20.0276 ppm centered at δ1H = 4.692 ppm. Hard proton 90° pulses were typically calibrated at 9.6W with an average value of 9.7 µs across the samples studied. Data acquired for D_2O-dissolved samples were acquired over a frequency width equivalent to 12.0166 ppm (acquisition time: 2.27 s).

Two-dimensional (2D) NMR data sets were acquired as follows for samples dissolved in D_2O. Proton-only data were acquired over frequency widths equivalent to 12 ppm (7194 Hz) in both F2 and F1 centered at an offset of δ1H = 4.702 ppm. 2D DQF-COSY data (pulse program: cosydfphpr) were acquired with 16 transients for each of 1024 States - TPPI t1 increments for acquisition times (aq) of 285 ms (ω2) and aqmax = 71 ms (ω1) and a total data accumulation time of 10 hrs. 2D TOCSY data (pulse program: dipsi2phpr) were acquired into 2048 data points (ω2 acquisition time = 142 ms) with eight transients for each of 512 States - TPPI t1 increments (aqmax ω1 = 35.4 ms) using a spin-lock time of 70 ms. The

same conditions were used to acquire 2D NOESY data (pulse program: noesyphpr) with mixing times ranging from 100 ms to 250 ms (four mixing times total). 2D [^{1}H, ^{13}C] correlation data were acquired using echo/anti-echo-TPPI data acquisition schemes with sensitivity improvement, with (pulse program: hsqcedetgpsisp. 2) and without (pulse program: hsqcetgpsisp.2) DEPT-editing and without/with non-uniform sampling (NUS), respectively. NUS data were acquired with 25% sampling of 512 t1 increments into 2048 data points for frequency widths equivalent to 10 ppm (F2) and 170 ppm (F1) with 128 transients per t1 increment for a total data accumulation time of 10 h. DEPT-edited HSQC data were acquired with a traditional acquisition mode using the same conditions and 64 transients per t1 increment for a total data accumulation time of 20 hrs. For samples dissolved in 90% H_2O, 2D NOESY data were acquired with excitation sculpting for solvent suppression (pulse program: noesyesgpph) using State-TPPI for 1024 t1 increments and 4096 data points over F2 and F1 frequency widths equivalent to 25 ppm centered at the solvent frequency resonance. Data were acquired with 16 transients per t1 increment with a relaxation delay of 7 s between transients for a total data accumulation time of 33 h. Mixing times were defined as 180 and 250 ms. All data were processed according to established NMR data processing protocols and transferred into SPARKY[5] software for data assignment and reduction in preparation for molecular structure calculations.

Molecular dynamics methods

Preliminary Molecular Dynamics

Preliminary molecular dynamics for distance restraint calculations of the **PA4•ODN4** complex was run in explicit solvent using the SANDER module of AmberTools16.[6] The starting model of **ODN4** was generated using the UCSF CHIMERA 1.12 program.[7] The structures of **PA4•ODN4** complex was generated based on an X-ray-derived structure of a cyclic polyamide•dsDNA complex as the starting structure (PDB ID 3OMJ).[8] Input files (prmtop and inpcrd) were generated using the nucleic acid force field ff99bsc1 and GAFF for **ODN4** and ligands, respectively. 21 Na^{+} ions for the complex were added to neutralize the negative charge of the phosphate groups. An octahedral box with outer edges of approximately 10.0 Å of TIP3P water was placed around the structures. A 10 Å cut-off for non-bonded interactions was applied with the Particle Mesh Ewald (PME) method to account for long range

electrostatic interactions. Initial minimizations of 1000 steps (500 steps of steepest descent and 500 steps of conjugate gradient minimization) were performed keeping the solute fixed with a 500 kcal/mol·A force constant. The entire system was then minimized over 2500 steps. A first MD run of 10000 steps was performed (20 ps) using the SHAKE algorithm. The initial temperature of 0 K was brought gradually up to 300 K and kept constant using Langevin dynamics and keeping the solute fixed with weak restraints (10 kcal/mol·A). The RST file was then used for a second MD run of 50000 steps (100 ps) removing the restraints. A snapshot was recorded every 100 steps to generate the trajectory file. RST files were checked for general consistency with the NOE data and used for distance calculations

NMR Distance Restraints

In all cases, the distance restraints were obtained from 2D [^{1}H-^{1}H] NOESY (250, 200, 150, and 100 ms mixing times). NMR-FAM SPARKY was used for the assignment process.[5] Non-overlapping peaks were integrated using the Gaussian fit to generate the intensity file for MARDIGRAS.[9] Through CORMA.IN, the RST files obtained from preliminary molecular dynamics were converted into pdb format to be used with MARDIGRAS (using an isotropic model and relative error of 10%). The correlation times used were roughly established in each case checking MARDIGRAS calculation outputs from 1 ns to 8 ns. Absolute unnormalized noise was defined as a fraction of the smallest peak that could be integrated in the spectrum. The methyl jump 3 model was used. MARDIGRAS was run for 50 cycles. The resulting ".dst" files were compiled with RAND-RESTR to generate an average distance file. These coordinates were converted to restraints for input to AMBER using M2AHOMO and filtering out selected out-of-range distances. The force constants applied were 10 kcal/mol·A^2 for lower and upper bounds. The width of the parabola was set to be 2 Å in all cases. Calculations were repeated at the beginning of each molecular dynamics cycle as reported below.

NMR-restrained molecular dynamics simulations

Initial molecular dynamics were run using the Generalized-Born implicit solvent model. After initial minimization (500 steps, 250 steepest descent, 250 conjugated gradient), 100 ps MD were run at constant temperature. NMR restraints were gradually increased over the first 20 ps and kept constant for

the remaining 80 ps. The output files were error checked, and the RST file was used for a second cycle of MARDIGRAS distance calculations. The new restraints were applied this time using the explicit solvent model and following the same protocol as above for the preliminary molecular dynamics. The RST file was then used as for the last cycle of MARDIGRAS and the calculated restraints were applied for the third MD simulation. A production run of 1 ns was obtained. Clustering of the last 800 ps was carried out using UCSF Chimera 1.12 to produce 11 representative structures which were each minimized and deposited in the Protein Data Bank (PDB ID **6GZ7**). An average minimized structure was obtained from the entire ensemble of (minimized) clusters using the CPPTRAJ module of AmberTools16. Utilizing this average minimized structure, and removing the polyamide through Chimera, DNA structural groove analysis along with helical parameters were calculated using both Curves+ and X3DNA software to complement each other.[10,11] Bond distances, calculations, and the generation of structural figures were performed on the average minimized structure using UCSF Chimera.

4.0 References

[1] G. Padroni, J. A. Parkinson, K. R. Fox, G. A. Burley, *Nucleic Acids Res.* **2018**, *46*, 42–53.

[2] M. . El Hassan, C. . Calladine, *J. Mol. Biol.* **1998**, *282*, 331–343.

[3] E. E. Baird, P. B. Dervan, *J. Am. Chem. Soc.* **1996**, *118*, 6141–6146.

[4] M. Wetzler, D. E. Wemmer, *Org. Lett.* **2010**, *12*, 3488–3490.

[5] W. Lee, M. Tonelli, J. L. Markley, *Bioinformatics* **2015**, *31*, 1325–1327.

[6] D. A. Case, D. S. Cerutti, T. E. I. Cheatham, T. A. Darden, R. E. Duke, T. J. Giese, H. Gohlke, A. W. Goetz, D. Greene, N. Homeyer, et al., *Univ. California, San Fr.* **2017**, AMBER 2017, University of California, San Francisc.

[7] E. F. Pettersen, T. D. Goddard, C. C. Huang, G. S. Couch, D. M. Greenblatt, E. C. Meng, T. E. Ferrin, *J. Comput. Chem.* **2004**, *25*, 1605–1612.

[8] D. M. Chenoweth, P. B. Dervan, *J. Am. Chem. Soc.* **2010**, *132*, 14521–14529.

[9] B. A. Borgias, T. L. James, *J. Magn. Reson.* **1990**, *87*, 475–487.

[10] C. Blanchet, M. Pasi, K. Zakrzewska, R. Lavery, *Nucleic Acids Res.* **2011**, *39*, W68–W73.

[11] X.-J. Lu, W. K. Olson, *Nucleic Acids Res.* **2003**, *31*, 5108–5121.
